# Achieving Robust and High-Transmittance Bioceramics with Minimal Organic Additives via a Supervariate Strategy

**DOI:** 10.1021/acsnano.6c04208

**Published:** 2026-08-14

**Authors:** Jie Zhou, Jiahua Liu, Hong Yue, Chao Fu, Yunchen Long, Xinxue Tang, Jian Lu, Yang Yang Li

**Affiliations:** † Department of Materials Science and Engineering, 53025 City University of Hong Kong, Hong Kong, China; ‡ Hong Kong Branch of National Precious Metals Material Engineering Research Centre, 53025 City University of Hong Kong, Hong Kong, China; § Center of Super-Diamond and Advanced Films (COSDAF), 53025 City University of Hong Kong, Hong Kong, China; ∥ Centre for Advanced Structural Materials, City University of Hong Kong Shenzhen Research Institute, Shenzhen, 518057, China; ⊥ Department of Mechanical Engineering, 53025 City University of Hong Kong, Hong Kong, China

**Keywords:** amorphous, bioceramics, toughness, supervariate, nanofusion, room temperature treatment, transparency

## Abstract

A fundamental dilemma in synthetic ceramic composites is that incorporating polymers to enhance toughness sacrifices hardness and often necessitates high organic content for effective integration. Nature solves this differently, building strong, robust minerals like nacre and tooth enamel with no more than 10% organic content—a contrast pointing to alternative material design principles. Here, we report a “supervariate” strategy to produce strong, robust biomineral–organic hybrids: flowable minerals that are multi-ionic, hydrated, and amorphous require only minimal organic additives to achieve thorough interfacial blending and nanofusion when pressed in an aqueous, room-temperature environment. The resulting composites deliver uncompromised hardness (1.6 GPa), excellent tensile ductility (10%), compressive toughness (95 MJ m^–3^), and high transmittance (78%) for millimeter-thick samples. This work provides an energy-efficient pathway to functional mineral monoliths that are strong, ductile, lightweight, and damage-tolerant and offers insights into biomineralization mechanisms.

## Graphical abstract

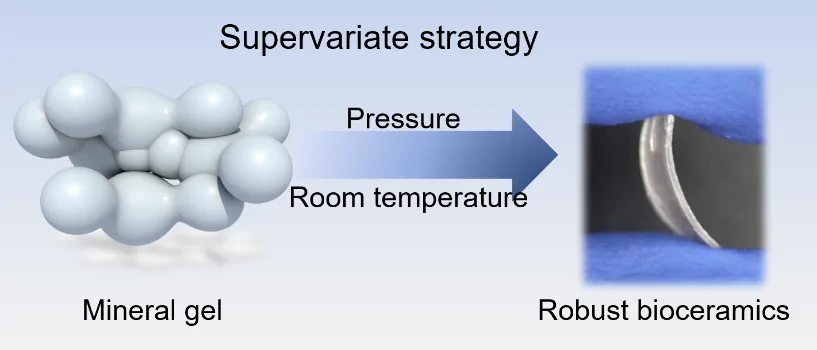

Ceramic materials are indispensable in every sector of modern society due to their exceptional hardness, thermal stability, and chemical resistance.^1–4^ However, their intrinsic brittleness and low toughness severely limit their reliability in load-bearing and impact-resistant applications.^5–7^ To address this issue, researchers have long been dedicated to enhancing the mechanical properties of composite materials by incorporating soft polymer phases into ceramic matrices.^8–10^ Despite enhanced ductility, these hybrid materials typically require high organic content (often exceeding 50 wt %) to ensure processability and toughness.^11,12^ Such a high polymer content inevitably results in a significant loss of ceramics’ inherent properties, such as hardness and thermal stability.

In stark contrast, biological systems master the art of forging high-performance structural materials in aqueous environments at body temperature with only a small amount of organic additives.^13,14^ Biogenic minerals, such as nacre, tooth enamel, and sea urchin spines, contain 90–95 wt % inorganic components, yet exhibit retained hardness together with improved ductility and toughness.^6,15–17^ Notably, biogenic minerals are often observed to evolve from hydrated amorphous precursors, such as amorphous calcium carbonate (ACC) or amorphous calcium phosphates (ACP).^18–20^ This has inspired extensive biomimetic efforts. However, achieving high mechanical performance in biomineral/organic hybrids with minimal organic additives remains a formidable challenge.

In this study, to address this challenge, we adopt a “supervariate” strategy which is characterized by the richness in ionic species, hydration traits, and disordered structures.^21,22^ In this state, multiple coprecipitating ions and diverse hydration species collectively generate a disordered and kinetically frustrated mineral network, which stabilizes the amorphous phase and enables pressure-assisted fusion under mild aqueous conditions. This facile and versatile strategy enables the acquisition of a flowable mineral gel phase,^23,24^ which is pivotal for ensuring its thorough integration with organic macromolecules, thereby facilitating the fabrication of homogeneous and dense hybrids. Specifically, a “supervariate” mineral-based precursor was synthesized by mixing Mg^2+^, Ca^2+^, carbonate, and phosphate ions with a small amount of poly­(acrylic acid) (PAA). Subsequent cold-pressing at room temperature triggers a consolidation process, resulting in extraordinarily strong and robust (a hardness of 1.6 GPa, a compressive plasticity of 36% and toughness of 95 MJ m^–3^) bioceramics with an organic content no more than 10 wt %. Moreover, the fabricated bioceramics exhibit high optical transmittance, reaching 78% for a millimeter-thick sample, suggesting good structural integrity and homogeneity resulting from the effective fusion of the mineral and organic components.

## Results and Discussion

### Material Design

We employed a multi-ionic biomimetic mineralization approach, starting with a mixed solution of calcium and magnesium salts in the presence of PAA (Figure 1a). Upon the introduction of carbonate and phosphate sources, the PAA chains regulated the nucleation and growth of the inorganic phase, leading to the formation of stabilized amorphous calcium–magnesium carbonate/phosphate (PAA-MgACCP) suspension^21^ (Figure S1a). These hybrid minerals were collected via centrifugation to form a viscous mineral gel (Figures S1b and S2). X-ray diffraction confirmed the amorphous character of gels prepared with different PAA contents (Figure S3). The semiwet gel was then consolidated by pressure-driven fusion into dense monolithic tablets. The optimized PAA-MgACCP tablets display high optical transmittance and bending deformability (Figure 1c), and cross-sectional SEM images show a compact and fused internal morphology. The proposed interfacial structure is shown in Figure 1b: carboxylate groups on PAA coordinate Ca^2+^ and Mg^2+^ at the mineral surface, while retained water and ionic interactions help bridge the organic and inorganic components.^25^ Electrostatic interactions between the mineral anions (CO_3_^2–^, PO_4_^3–^) and the cationic species further stabilize the network.

**Figure 1:**
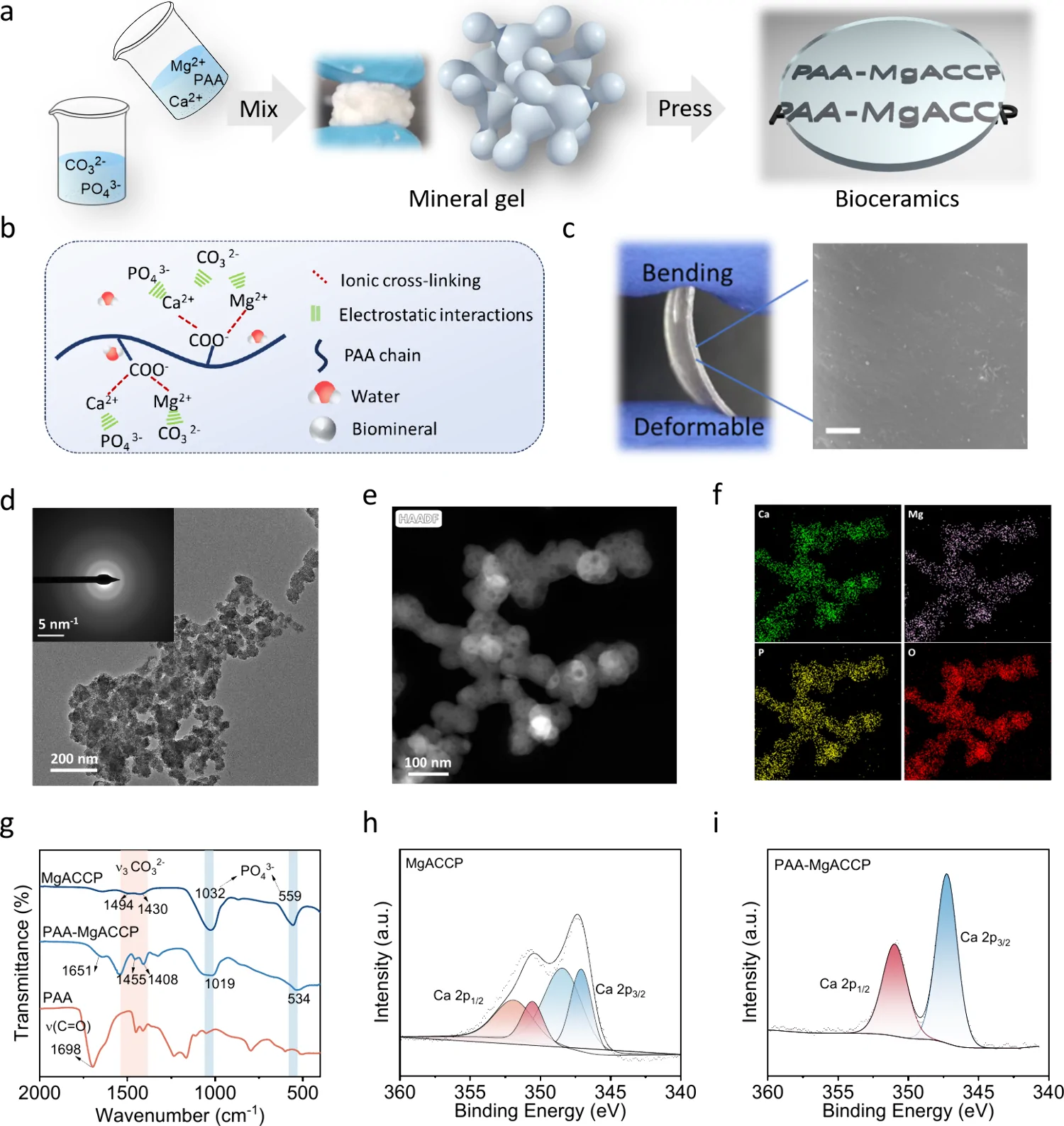
Biomimetic synthesis and structural characterization of the PAA-MgACCP bioceramics. (a) Schematic illustration of the preparation process of PAA-MgACCP. (b) Schematic diagram of the structure of mineral ceramics. (c) Photograph and the cross-section SEM images of PAA-MgACCP tablets. Scale bar, 5 μm. (d) TEM image showing that the gel consists of nano spherical particles, with the selected area electron diffraction (SAED) pattern displayed in the top-left corner. (e) Scanning TEM image and (f) elemental mapping showing the uniform distribution of Ca, Mg, P, and O. (g) FTIR spectra of PAA, MgACCP, and PAA-MgACCP. (h, i) XPS spectra of calcium in MgACCP and PAA-MgACCP.

Transmission electron microscopy (TEM) shows that the PAA-MgACCP gel is composed of nanospherical aggregates (Figure 1d). The corresponding selected-area electron diffraction pattern contains a diffuse halo, supporting the amorphous character of the precursor. Particle-size statistics obtained from SEM images are summarized in Figure S4. As shown in Figure 1e and 1f, scanning transmission electron microscopy (STEM) imaging combined with elemental mapping technology confirmed the uniform distribution of the four constituent elements in the PAA-MgACCP gel.^21,26^ For comparison, we also analyzed the morphology and structure of MgACCP (Figures S5 and S6). The Fourier transform infrared (FTIR) spectrum (Figure 1g) shows a characteristic C=O stretching vibration peak at 1698 cm^–1^, which is attributed to the abundant carboxyl (−COOH) groups in the PAA chains.^27^ After incorporation into MgACCP, this band becomes weaker and broader, indicating coordination between metal cations and carboxyl groups.^28^ In MgACCP, the characteristic FTIR peaks of the asymmetric stretching vibration (v_3_) of CO_3_^2–^ appear at 1494 cm^–1^ and 1431 cm^–1^, while the vibration peaks of PO_4_^3–^ appear at 1032 cm^–1^ (v_3_ P–O stretching) and 559 cm^–1^ (v_4_ O–P–O bending).^29^ Upon the addition of PAA, these peaks shift to 1455/1408 cm^–1^ (CO_3_^2–^) and 1019/534 cm^–1^ (PO_4_^3–^), which can be attributed to the competition between the COO^–^ of PAA and the mineral ions for coordination sites. X-ray photoelectron spectroscopy (XPS) further reveals the effect of PAA on the local Ca coordination environment (Figure 1h and (i). Compared with pristine MgACCP, PAA-MgACCP exhibits a sharper and more symmetric Ca 2p envelope, with the main Ca 2p3/2 and Ca 2p1/2 peaks located at 347.35 and 350.82 eV, respectively. The narrowed peak profile indicates a reduced distribution of local Ca environments in the amorphous mineral matrix. This result suggests that the carboxylate groups of PAA coordinate with Ca^2+^ and help regulate the mineral-polymer interface, leading to a more homogeneous local coordination network in PAA-MgACCP. A more detailed fit analysis and the full-spectrum data are shown in Figure S7.

### Pressure-Driven Fusion Regulation

The pressure-driven preparation process is shown in Figure 2a. Owing to the viscoelastic and moldable nature of the gel state, it can be pressed into tablets with different shapes, including clover-like, cylindrical, circular, and rectangular specimens (Figure 2b). Consolidation is achieved without high-temperature sintering, relying instead on dehydration and mineral fusion.^30^ As shown in the SEM images and the corresponding binarized SEM images in Figure 2c, distinct nanoparticles and their boundaries are observed at *P* < 50 MPa. When the pressure increases to 200 and 500 MPa, extensive coalescence of particles occurs. As *P* further increases to 800 MPa, the spherical features become largely indistinguishable, indicating extensive particle coalescence and formation of a continuous compact. EDS mapping confirms the homogeneous distribution of all elements (Figure S8). Optical transparency also improves with increasing pressure, as the reduction of pores and particle-boundary scattering allows text beneath the tablet to be seen more clearly. Without pressure-driven consolidation, the evaporation of the solvent induced significant volume shrinkage and internal stress, eventually leading to the fragmentation of the gel (Figure S9).

**Figure 2:**
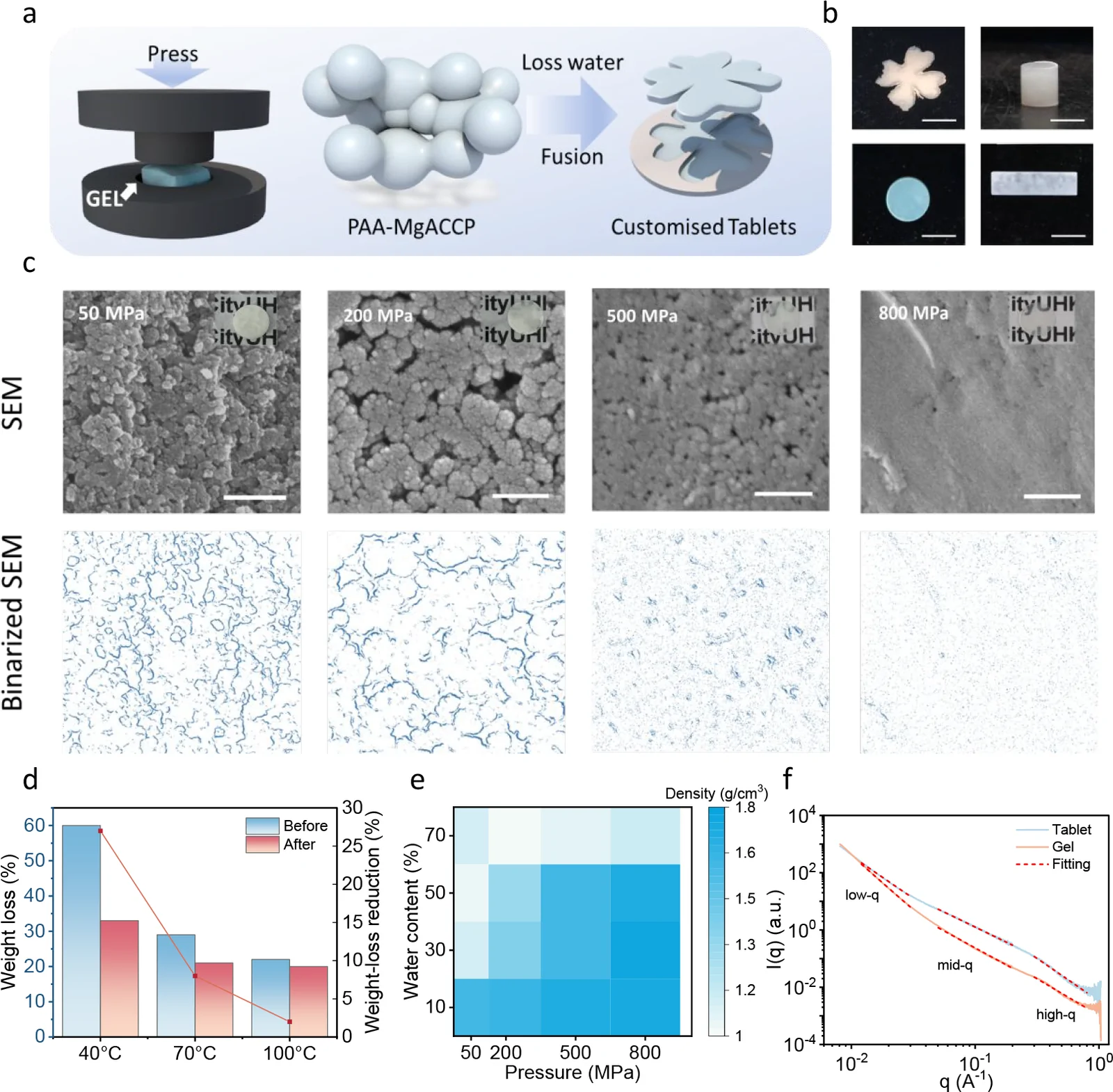
Preparation and characterization of biomimetic pressure-driven PAA-MgACCP tablets. (a) Schematic diagram of the tablet preparation process using a tableting mold. (b) PAA-MgACCP is processed into four-leaf clover leaves, cylinders, circular discs, and rectangles, enabling pattern formation. Scale bar, 1 cm. (c) SEM surface images of PAA-MgACCP tablets compressed at 50, 200, 500, and 800 MPa, along with their corresponding binarized processed images. Blue indicates unconsolidated areas; white indicates fused areas. Scale bar, 500 nm. (d) Weight-loss reduction before and after compaction. (e) Density map of mineral tablets as a function of compression pressure and water content. (f) SAXS profiles of the gel and pressed tablet, plotted on a log–log scale. The dashed lines show power-law fitting.

To clarify the role of water in the particle fusion process, the water contents of PAA-MgACCP gel powders dried at different temperatures are analyzed by TGA (Figures 2d and S10). The water content is calculated from the mass loss in the 50–250 °C range.^30^ At a low drying temperature of 40 °C, a large amount of water remains in the gel, leading to excessive fluidity during compression. As a result, the precursor readily migrates within the mold and flows out from the mold edges (Figure S10d). In contrast, insufficient water content hinders particle rearrangement and uniform interparticle contact, resulting in rough and nonuniform tablet surfaces (Figure S10f). Among the three drying conditions, powders dried at 70 °C for 2 h exhibit the best formability, corresponding to a water content of approximately 30%.^31,32^ The tablet density ranged from approximately 1.0 to 1.8 g/cm^3^, with the highest densification (1.76 g/cm^3^) achieved at 30% water content under 800 MPa compression (Figure 2e). The tablet density is lower than that of most dense inorganic ceramics,^33,34^ which can be attributed to the incorporation of additional bound water facilitated by the presence of Mg^2+^ and PAA. XRD patterns show that the pressed tablets remain amorphous across the investigated pressure range (Figure S11). This amorphous state is retained after six months of aging. SAXS reveals pressure-driven nanoscale reorganization from the precursor gel powder to the pressed tablet (Figure 2f). Power-law fitting in three q ranges shows that α decreases from 3.79 to 2.96 in the low-q region, indicating reduced large-scale heterogeneity and a more compact aggregate after pressing. In the mid-q region, α remains below 3 (2.36 to 2.09), consistent with a disordered fractal-like network. In the high-q region, α increases from 2.56 to 2.97, suggesting reorganization of local density fluctuations and interfacial structures. Porod analysis (Figure S12) shows no clear plateau, indicating diffuse nanoscale interfaces. Together, these results support dehydration-assisted compaction and interfacial fusion during room-temperature pressing.

### Optical and Mechanical Properties of Bioceramics

The optical transmittance of PAA-MgACCP tablets is shown in Figure 3a. The average visible transmittance (400–800 nm) decreases from 87% to 82%, 78%, 60%, and 54% as the PAA content decreases from 10 to 0 wt %. A higher PAA content can more effectively fill the voids between particles, creating a smoother mineral-polymer interface, thereby reducing scattering and improving transparency (Figure S13). To further evaluate the optical scattering behavior, haze measurements were conducted using a selected 5 mm aperture, as shown in Figure S14. Raman spectroscopy is used to probe the interaction between the amorphous mineral phase and PAA (Figure 3b). With increasing PAA content from 2 to 10 wt %, the intensity of the CH_2_ asymmetric stretching vibration gradually increases,^35^ while the characteristic symmetric stretching vibration peak of HPO_4_^2–^ progressively weakens. This trend is primarily attributed to the weak acidity and strong coordination capability of PAA. Additionally, peak deconvolution analysis of the O–H stretching region (3000–3700 cm^–1^) indicates that the incorporation of PAA significantly alters the hydrogen-bonding environment of bound water in the tablets (Figure S15). The dehydration process of PAA-MgACCP tablets no longer exhibits the typical three-stage water loss behavior compared to pure MgACCP tablets,^36,37^ but instead shows a more continuous mass loss with overlapping temperature zones (Figure S16). PAA exhibits good hydrophilicity, with polymer hydration water loosely adsorbed between segments or near carboxyl groups. This alters pore structure and water diffusion pathways, thereby reducing the differences in dehydration rates across various stages (Figure S17).

**Figure 3:**
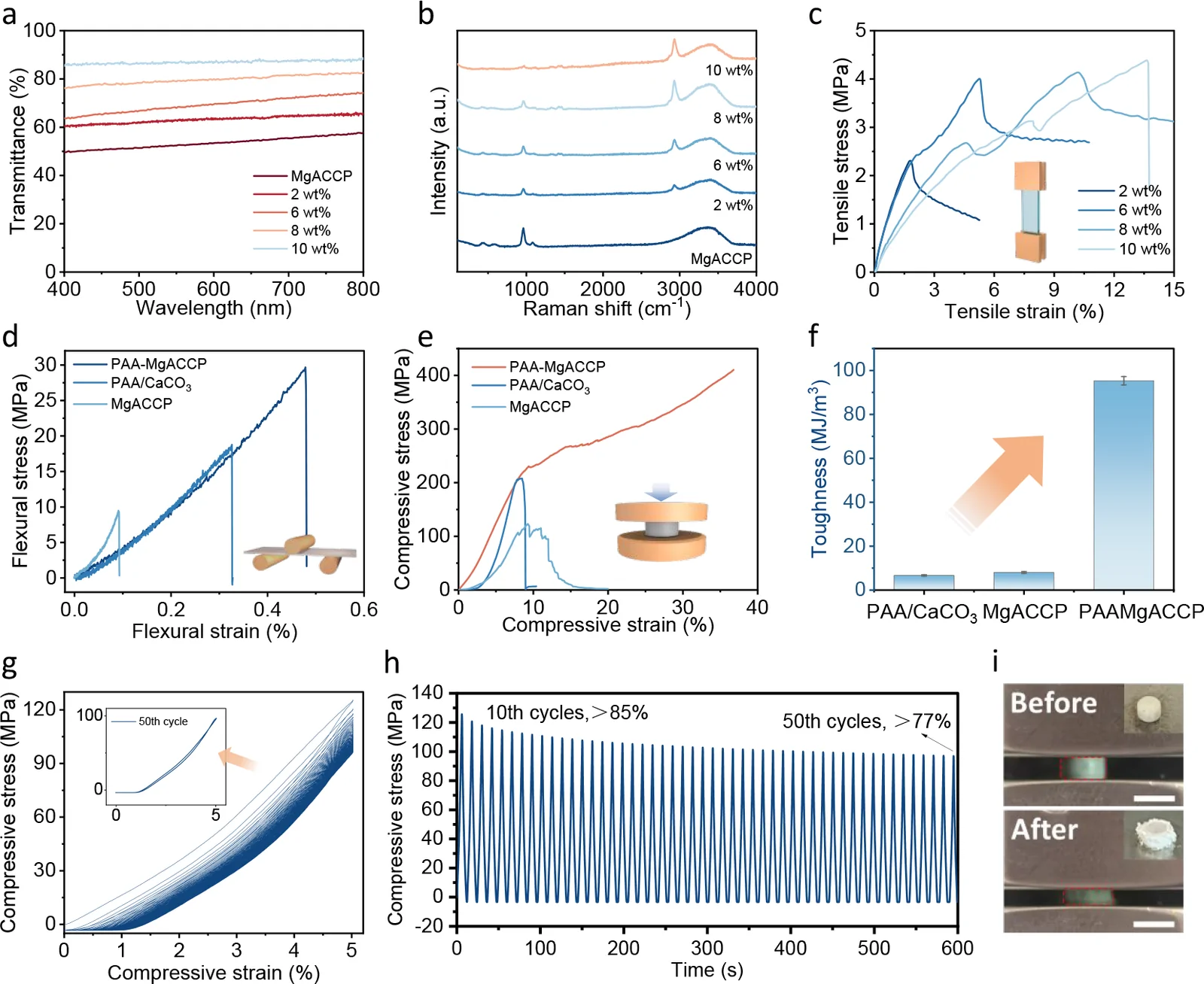
Optical and mechanical properties analysis of PAA-MgACCP tablets. (a) Transmittance spectra in the visible light region (400–800 nm) for tablets with different PAA contents. (b) Raman spectra of PAA-MgACCP tablets. (c) Tensile stress–strain curves of PAA-MgACCP tablets with different PAA contents (2, 6, 8, and 10 wt %). (d) Flexural stress–strain curves converted from three-point bending tests of PAA-MgACCP, PAA/CaCO_3_, and MgACCP. (e) Uniaxial compressive stress–strain responses of PAA-MgACCP, PAA/CaCO_3_, and MgACCP tablets. (f) Comparison of apparent compressive energy absorption density for PAA/CaCO_3_, MgACCP, and PAA-MgACCP. (g) Cyclic compressive stress–strain curves of PAA-MgACCP over 50 loading–unloading cycles up to 5% strain (inset: 50th cycle). (h) Time-dependent peak compressive stress of PAA-MgACCP during 50 consecutive compression cycles. (i) Photographs of a PAA-MgACCP tablet before and after uniaxial compression (scale bar, 5 mm).

The uniaxial tensile stress–strain behavior of PAA-MgACCP tablets is shown in Figure 3c. The 2 wt % sample fractures at 1.8% strain and 2.3 MPa. At 6 wt % PAA, the composite reaches a peak stress of ∼4.0 MPa at 5.8% strain, exhibiting a high Young’s modulus of 169.0 MPa. Higher contents (8 and 10 wt %) display distinct stress-drop and rehardening events. Ultimately, the 10 wt % sample achieves 13.1% ductility and ∼ 4.3 MPa maximum stress, while its modulus drops to 64.3 MPa. This is attributed to the organic phase tending toward greater continuity and local enrichment. Microstructural analysis revealed the presence of multiscale cracks, tortuous crack paths, and irregular fracture zones on the fracture surface (Figure S18). These observations are consistent with a synergistic energy dissipation mechanism, in which the presence of PAA helps the material sustain deformation through chain-mediated buffering and interfacial coordination. It is worth noting that due to the inherent brittleness of PAA/CaCO_3_ and MgACCP, tensile testing cannot be performed on these materials individually (Figure S19). Based on the combined evaluation of optical transmittance and tensile strength, the sample containing 6 wt % PAA-MgACCP is selected for subsequent tests (Figure S20 and Table S1). The results of the three-point bending test indicate that the PAA-MgACCP specimen exhibited high load-bearing capacity and a certain degree of energy absorption during loading,^38,39^ with a flexural strength of 30.7 MPa and a flexural energy absorption density of 74 kJ m^–3^ (Figures 3d and S21).

Figure 3e compares the uniaxial compressive responses of crystalline PAA/CaCO_3_, amorphous MgACCP, and PAA-MgACCP under the same fixed geometry. PAA/CaCO_3_ and MgACCP fail at low strains of 8.2% and 9.4%, respectively, accompanied by abrupt stress drops typical of brittle fracture. In contrast, PAA-MgACCP shows plateau like deformation and strain hardening, sustaining large compressive strain without catastrophic disintegration. Under this fixed testing geometry, PAA-MgACCP reaches an ultimate compressive strength above 400 MPa, and the apparent compressive energy absorption density calculated from the stress–strain curve reaches 95.3 MJ m^–3^ at 36% strain (Figure 3f). Postcompression SEM observations reveal a gradual damage process involving local compaction, structural reorganization, and distributed microcrack accumulation, while the overall specimen remains continuous (Figure S22). Macroscopic images further show that PAA-MgACCP retains its disc-like integrity after compression, whereas MgACCP and PAA/CaCO_3_ undergo more severe fracture and fragmentation (Figures 3 **i and** S23). The superior response of PAA-MgACCP over both controls indicates that its deformation tolerance arises from the synergistic coupling between the amorphous MgACCP network and PAA-mediated interfacial regulation. The influence of specimen geometry is further evaluated using 1:1 height-to-diameter samples (Figure S24). Although the absolute compressive values decrease under this more conservative geometry, PAA-MgACCP still exhibits the highest compressive strength and energy absorption among the tested groups, confirming that the material ranking remains valid. The decrease in thicker specimens is attributed to increased processing-induced heterogeneity during pressure-driven dehydration and fusion. Cross-sectional SEM images further show that amorphous mineral fusion produces a smoother and less defective internal morphology than crystalline-phase compaction (Figure S25).

The cyclic compressive response behavior of the PAA-MgACCP specimen during 50 loading–unloading cycles at 5% strain is shown in Figure 3g. This test is used to evaluate cyclic compressive stability under repeated large-strain deformation. The peak stress decreases from 125.7 MPa in the first cycle to approximately 97.0 MPa after 50 cycles, corresponding to a retention of about 77%. This stress decay indicates progressive internal rearrangement and damage accumulation during cycling. Nevertheless, the specimen does not undergo catastrophic fracture or macroscopic disintegration, and the 50th cycle still retains a smooth stress–strain profile. Additional cyclic compression at 1% strain further shows that PAA-MgACCP retains 91% of its initial peak stress after 50 cycles (Figure S26). These results suggest that PAA-MgACCP maintains substantial load-bearing capacity under repeated large-strain compression. This cyclic response is associated with the PAA-mediated amorphous mineral network, where interfacial rearrangement, compaction, and distributed microdamage dissipate energy while delaying unstable fracture.^40–42^

### Structure–Property Relationship Analysis

The representative microindentation load-depth curves for MgACCP, PAA-MgACCP, and PAA/CaCO_3_ are shown in Figure 4a. At a given maximum load, both MgACCP and PAA-MgACCP exhibit much smaller penetration depths than PAA/CaCO_3_, indicating a substantially higher resistance to plastic indentation. Figure 4b quantitatively compares the indentation modulus and hardness extracted from these curves. PAA/CaCO_3_ shows the lowest modulus (11.35 GPa) and hardness (0.40 GPa), confirming that simple physical mixing of PAA with crystalline CaCO_3_ leads to substantial softening. MgACCP exhibits a markedly higher modulus (22.15 GPa) and hardness (1.51 GPa), consistent with a dense amorphous mineral network. It is noteworthy that despite containing organic components, PAA-MgACCP retains a higher modulus (23.20 GPa) and hardness (1.63 GPa) than pure MgACCP. This is attributed to the multiple strong interactions present in the fusion tablets. The representative microindentation load-depth curves of PAA-MgACCP with PAA content of 2–10 wt % are shown in Figure S27. A moderate PAA content (6 wt %) achieves the best balance of hardness and toughness. The Ashby diagram in Figure 4c compares the indentation modulus versus hardness relationship for PAA-MgACCP with representative hybrid polymers, hybrid ceramics, biominerals, and engineered materials (Table S2).^16,43–51^ PAA-MgACCP occupies a region that combines bone-like stiffness with substantially higher hardness than most polymer-rich hybrids. SEM revealed the indentation morphology of several tablets, as shown in Figure 4d-**f**. MgACCP exhibits a straight and smooth fracture pattern, whereas for crystalline PAA/CaCO_3_, fracture primarily occurs at crystal interfaces. In contrast, the main crack in PAA-MgACCP propagates along a meandering path, effectively dissipating energy while maintaining structural stability.^52,53^ In the *H* ^3^ */E* ^2^ *-H* Ashby plot, PAA-MgACCP exhibits both high hardness and a high *H* ^3^ */E* *^2^*,^54^ indicating a strongly suppressed tendency for indentation-driven irreversible deformation (Figure 4g).

**Figure 4:**
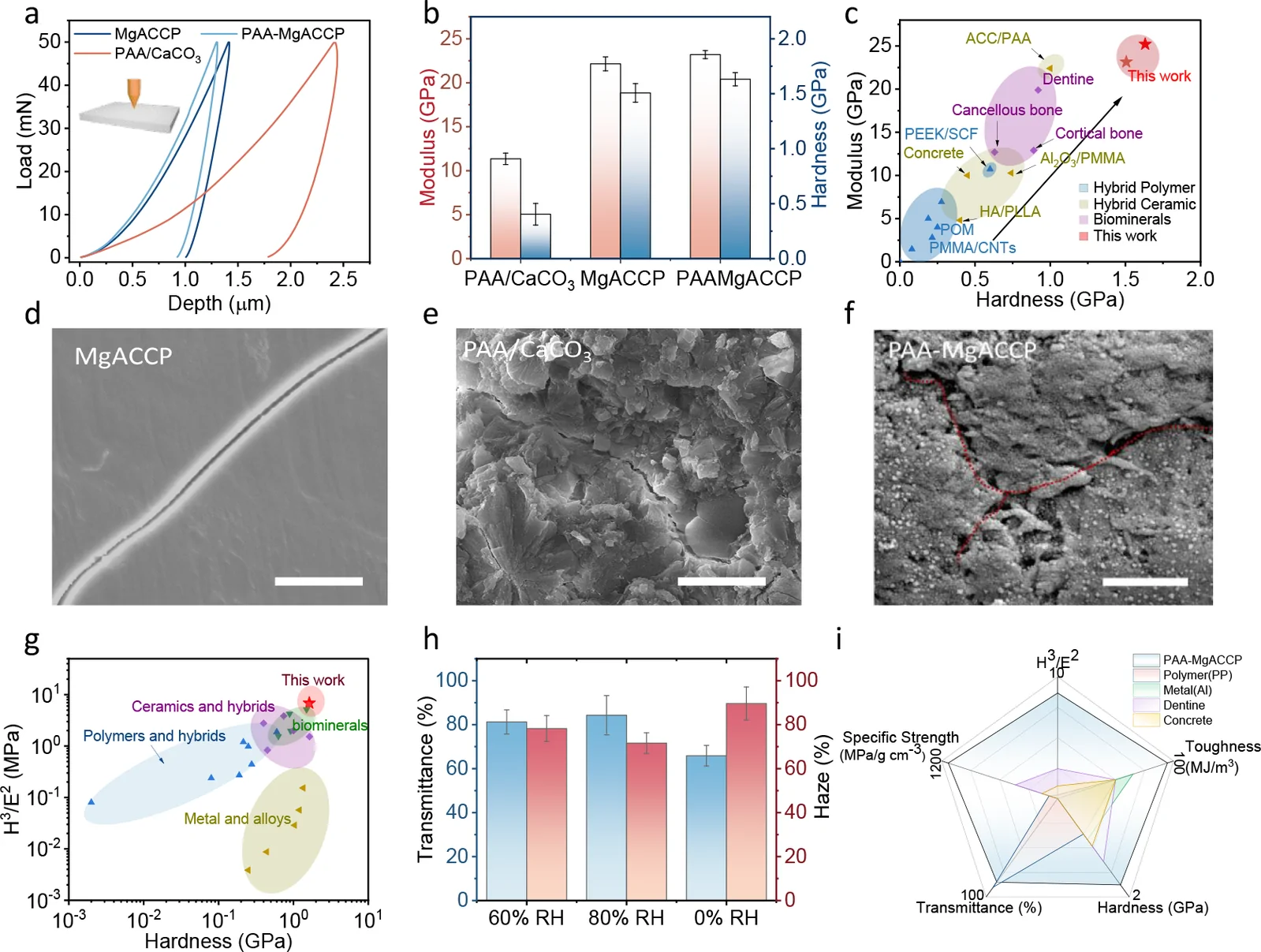
Mechanical performance and multifunctional properties of PAA-MgACCP tablets. (a) Representative microindentation load-depth curves for MgACCP, PAA-MgACCP and PAA/CaCO_3_ tablets, and (b) corresponding modulus and hardness values. (c) Ashby plot of E-H comparing this work (red star) with representative hybrid polymers, hybrid ceramics, and biominerals. (d-f) SEM images of crack propagation in MgACCP, PAA/CaCO_3_ and PAA-MgACCP tablets. Scale bar, 5 μm. (g) Ashby diagrams for *H* ^3^ */E* ^2^-H regarding composite engineering materials and this work. (h) Optical transmittance and haze of PAA-MgACCP tablets under 0%, 60%, and 80% humidity conditions. (i) Radar chart comparing PAA-MgACCP with polypropylene (PP), aluminum, dentine, and concrete in terms of hardness, toughness, *H* ^3^ */E* *^2^*, specific strength, and optical transmittance.

For practical applications, humidity resistance is essential for evaluating the reliability of advanced composites. We therefore investigated the mechanical and optical responses of the PAA-MgACCP under different relative humidity (RH) conditions (Figure 4h). At 60% and 80% RH, the composite maintains high transmittance of >80% and moderate haze. PAA-MgACCP bioceramics retains a high ultimate tensile stress of 4.0 and 3.9 MPa, respectively, with tensile strains of 9.5% and 8.5% (Figure S28). During direct flame exposure, the sample maintained its structural integrity and showed only limited combustion, whereas the organic-rich PAA control burned rapidly and was largely consumed (Figure S29 **)**. The flame-retardant performance of the material is primarily attributed to its high mineral content and low organic fraction (Table S3). As shown in Figure 4i and Table S4, the radar chart demonstrates that PAA-MgACCP exhibits high hardness, a large *H* ^3^ */E* *^2^* index, high specific strength, excellent toughness, and significant light transmittance compared to polymers (PP), metals (Al), bone, dentine, and concrete.^43,55–58^

## Conclusion

We have developed a scalable, energy-efficient, and room-temperature route to fabricate high-transmittance, strong, and robust bioceramics by employing a supervariate strategy. By cold-pressing a multi-ionic, hydrated, and amorphous gelatinous PAA-MgACCP, we achieved hybrids that reconcile the long-standing trade-off between hardness and ductility. The resulting bioceramic exhibits a high hardness of 1.6 GPa, an exceptional compressive toughness of 95 MJ m^–3^, and a tensile ductility of 10%, while exhibiting high transparency (78%). The supervariate strategy is a general approach applicable to diverse mineral systems for achieving strong, ductile ceramics with an ultralow organic content. Moreover, the inherent flame retardancy and optical clarity of these novel materials suggest great potential for tough glass, protective coatings, or aerospace devices where traditional plastics or brittle ceramics cannot be used. Beyond the promise of sustainable manufacturing of advanced ceramics, the powerful supervariate approach offers fresh insights into biomineralization mechanisms.

## Experimental Section

### Materials

Poly­(acrylic acid) (PAA, Mw = 250 000 g/mol, Aldrich), along with analytical-grade calcium chloride dihydrate (CaCl_2_·2H_2_O), magnesium chloride hexahydrate (MgCl_2_·6H_2_O), anhydrous sodium carbonate (Na_2_CO_3_), and disodium hydrogen phosphate (Na_2_HPO_4_), were all purchased from Sigma-Aldrich and used without further purification.

### Synthesis

A solution containing 0.025 mol of CaCl_2_ and 0.025 mol of MgCl_2_ was prepared in 100 mL of deionized water. Subsequently, an aqueous solution of PAA was added to this mixture, with the PAA mass fraction adjusted to 2–10 wt % relative to the total mineral content. The resulting mixture was magnetically stirred for 30 min and designated as Solution A. In a separate preparation, Solution B was obtained by dissolving equimolar amounts of Na_2_CO_3_ and Na_2_HPO_4_ in deionized water. Under continuous magnetic stirring, Solution B was slowly added into Solution A. Stirring was maintained for an additional 30 min after the complete addition. The resulting mixture was then washed five times via centrifugation with deionized water, followed by a final centrifugation step at 8000 rpm for 5 min to obtain the PAA-MgACCP gel. All experimental procedures were performed at room temperature (25 °C).

The semiwet gel, predried at 70 °C for 2 h, was subsequently transferred into molds with inner diameters of 6 mm, 8 mm, and 18 mm (Tianjin Jingtuo Instrument Technology Co., Ltd.). The gel was then compressed for 5 min using a hydraulic press (Power Team SPX manual pump, 10,000 PSI, P59 B type). Pressure was monitored in real-time via a digital pressure sensor (SBT710, Guangzhou Xinba Touch Technology Co., Ltd.). As the pressure was gradually increased to the target value, free water was progressively expelled from the gel. The thickness of the resulting tablets, ranging from 1 mm to 3 mm, was controlled by adjusting the amount of gel loaded into the mold prior to compression.

### Characterizations

The surface and cross-sectional morphologies of the samples were examined using a field-emission scanning electron microscope (FE-SEM, Thermo Fisher Apreo 2). Prior to SEM observation, the samples were sputter-coated with a thin layer of gold to enhance conductivity. Transmission electron microscopy (TEM) was performed on a FEI Talos F200S. Selected area electron diffraction (SAED) patterns were acquired to determine the crystallographic information on the specimens. High-angle annular dark-field (HAADF) imaging was conducted using a scanning transmission electron microscope (STEM) to obtain Z-contrast images and analyze elemental distribution at the nanoscale. X-ray diffraction (XRD) analysis was carried out using a D2 PHASER XE diffractometer with Cu Kα radiation (λ = 1.5406 Å). The diffraction patterns were recorded over a 2θ range of 10° to 80° with a step size of 0.01° and a scanning rate of 5°/min. Phase identification was performed by comparing the obtained patterns with standard JCPDS cards. Thermogravimetric analysis (TGA) was carried out using a PerkinElmer Simultaneous Thermal Analyzer (STA) 6000. The samples were heated from room temperature to 800 °C at a constant heating rate of 10 °C/min under a nitrogen atmosphere. The mass change as a function of temperature was recorded to evaluate the thermal stability and decomposition behavior of the samples. Small-angle X-ray scattering (SAXS) measurements (Xeuss 3.0) were performed with a scattering vector (q) range of 0.01 to 0.8 Å^–1^ to probe the nanoscale structure and pore characteristics of the materials. Fourier transform infrared spectroscopy (FTIR) was conducted on a PerkinElmer FTIR Spectrometer in the wavenumber range of 4000 to 200 cm^–1^. X-ray photoelectron spectroscopy (XPS) measurements were performed with monochromatic Al Kα radiation (hν = 1486.6 eV) to determine the surface chemical composition and oxidation states. The binding energies were calibrated using the C 1s peak at 284.8 eV as a reference. The optical transmittance spectra of the samples were measured using a Hitachi UH4150 UV–vis-NIR spectrophotometer in the wavelength range of 300 to 800 nm. Air was used as the baseline reference. Raman spectra were acquired using a WITec RAMAN alpha 300R confocal Raman microscope equipped with a 532 nm laser excitation source. The laser power was maintained at 10 mW to avoid sample damage, and the spectral range was set from 4000 to 200 cm^–1^ with an integration time of 1 s and 10 accumulations per spectrum.

Tensile tests were conducted on an Instron Series 3382 universal testing machine (UTM) at room temperature with a crosshead speed of 2 mm/min. The specimens were prepared with dimensions of 5 mm × 20 mm × 1 mm. The sample size for the compression test was a cylindrical specimen with a diameter of 6 mm and a thickness of 3 mm. The flexural properties of the samples were evaluated by three-point bending tests. Rectangular specimens with dimensions of 40 mm × 10 mm × 2.6 mm were tested using a support span of 26 mm. The flexural strength was calculated using the standard three-point bending equation. The area under the load–displacement curve was obtained by numerical integration, representing the energy absorption capacity during bending. Microhardness measurements were performed using a Fischer HM2000XY microhardness tester. A minimum of 5 indentations were made on each sample, and the reported values represent the average of these measurements. The experimental density was determined using the Archimedes method.

## Supplementary Material



## Notes

Rheological, structural, microscopic, spectroscopic, thermal, optical, and mechanical characterization; XPS fitting and SAXS analysis; humidity and flame-exposure tests; supplementary tables (PDF)
