# Supplementary material for: Achieving Robust and High-Transmittance Bioceramics with Minimal Organic Additives via a Supervariate Strategy
Source: ACS Nano. 2026 Aug 14;20(33):23234–43. doi: 10.1021/acsnano.6c04208 (PMC13523790; doi:10.1021/acsnano.6c04208)
Supplement: Supplementary file 1 [file nn6c04208_si_001.pdf]

# Supporting Information for

Achieving Robust and High-Transmittance Bioceramics with Minimal Organic Additives via Supervariate Strategy

Jie Zhou [a, b, c, d], Jiahua Liu [a, b, c, d], Hong Yue [a, b, c, d], Chao Fu [a, b, c, d], Yunchen Long [a, b, c, d], Xinxue Tang [a, b, c, d], Jian Lu [a, b, d, e] *, Yang Yang Li [a, b, c, d, e] *

[a] Department of Materials Science and Engineering, City University of Hong Kong, Hong Kong, China

[b] Hong Kong Branch of National Precious Metals Material Engineering Research Centre, City University of Hong Kong, Hong Kong, China

[c] Center of Super-Diamond and Advanced Films (COSDAF), City University of Hong Kong, Hong Kong, China

[d] Centre for Advanced Structural Materials, City University of Hong Kong Shenzhen Research Institute, Shenzhen 518057, China

[e] Department of Mechanical Engineering, City University of Hong Kong, Hong Kong, China

*Corresponding authors: jianlu@cityu.edu.hk; yangli@cityu.edu.hk

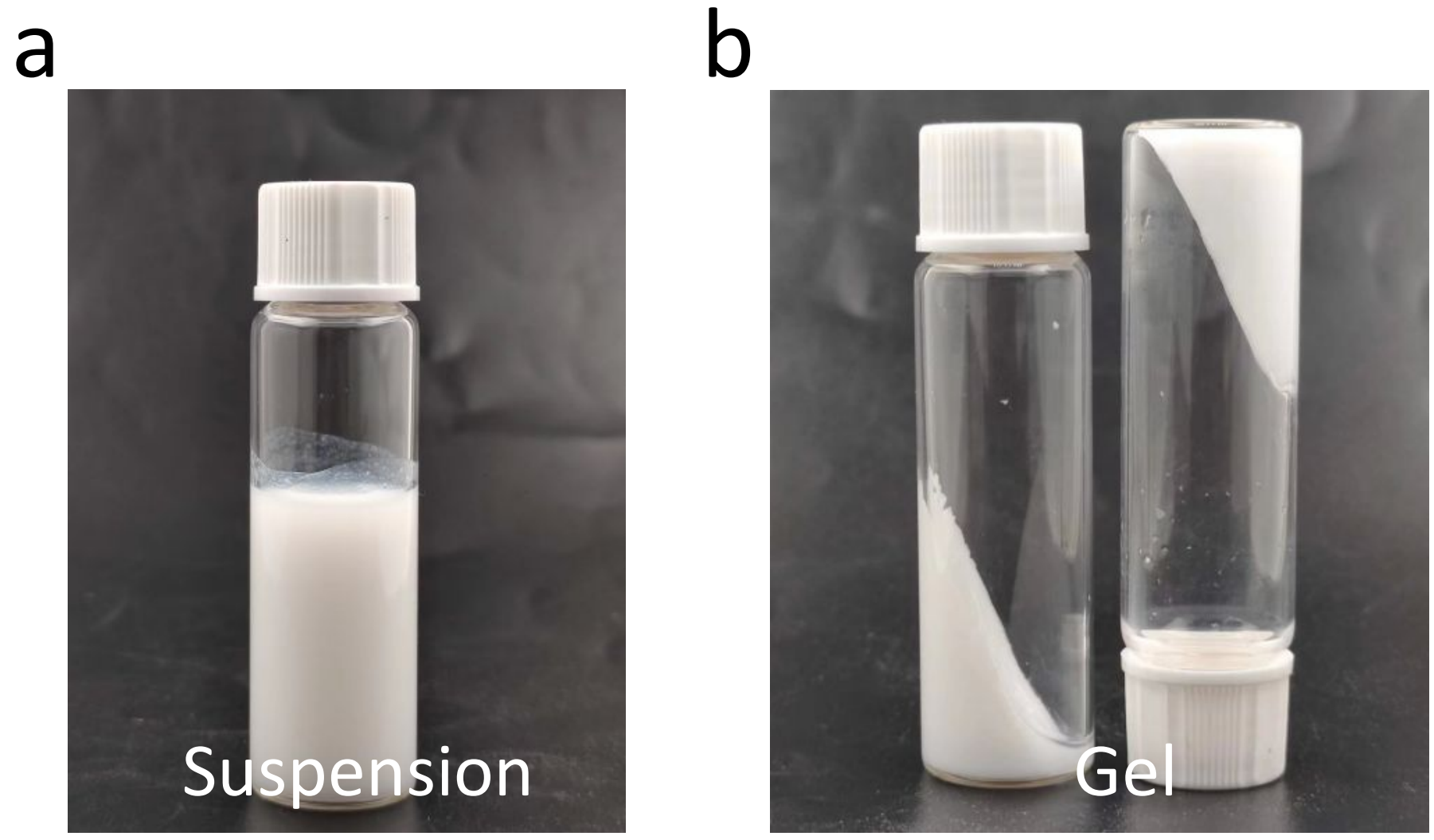


Figure S1. (a) Photographs of amorphous nanocluster suspensions and (b) amorphous mineral gels.

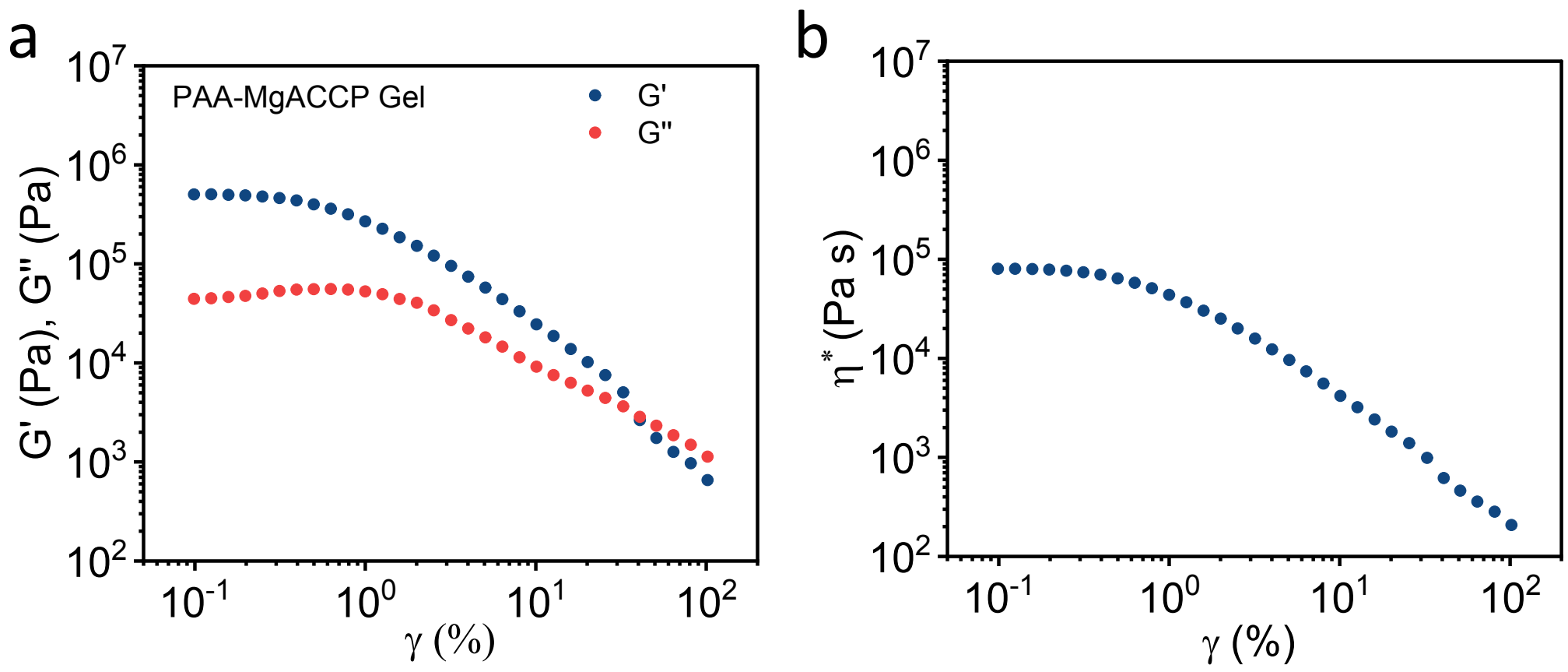


Figure S2. (a) Strain sweep measurements and (b) complex viscosity of PAA-MgACCP gel (from 0.1% to 100% strain) at 25 °C.

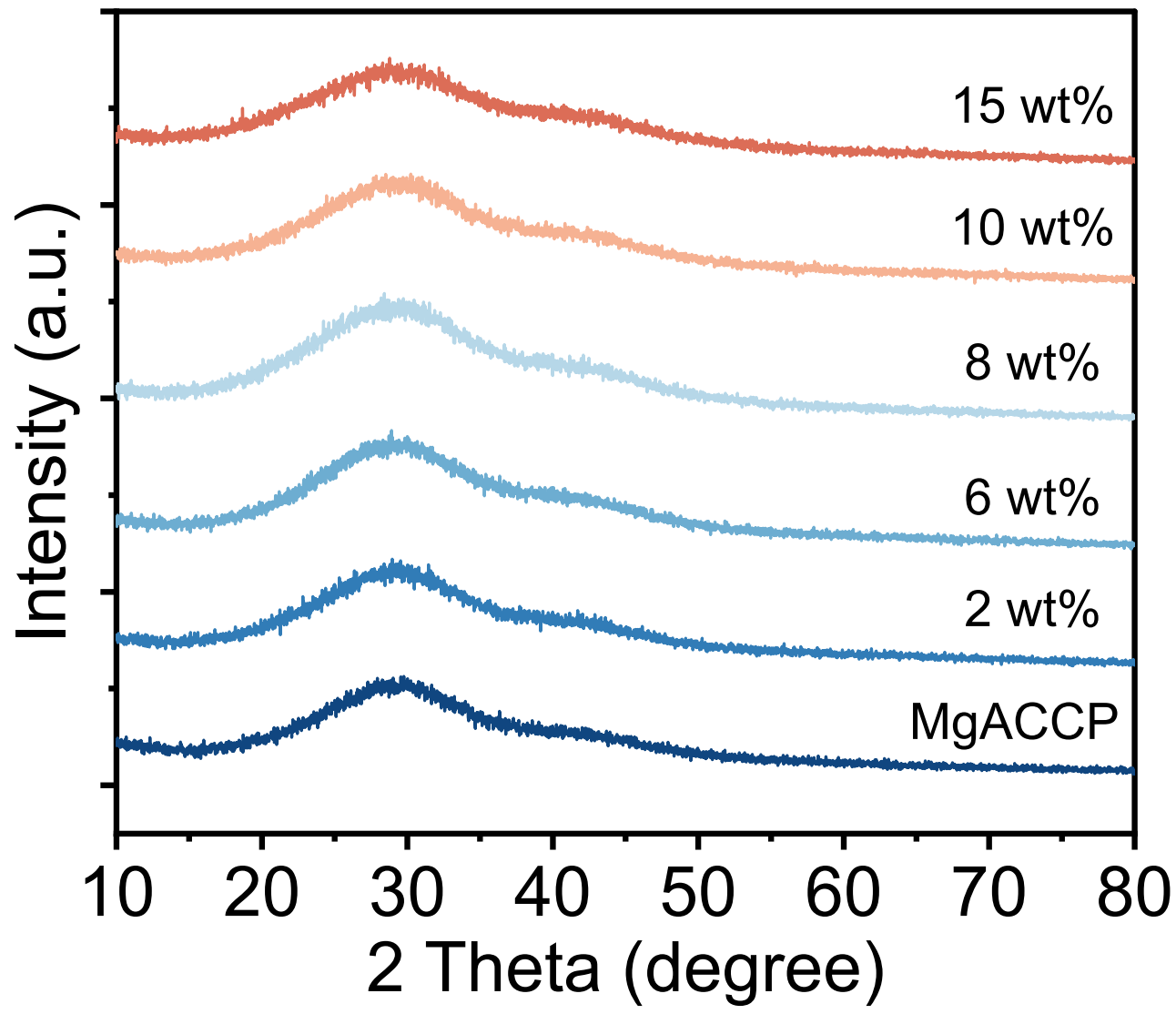


Figure S3. XRD spectra of MgACCP gel with 0, 2, 6, 8, 10 and 15 wt% PAA. All samples remain X-ray amorphous.

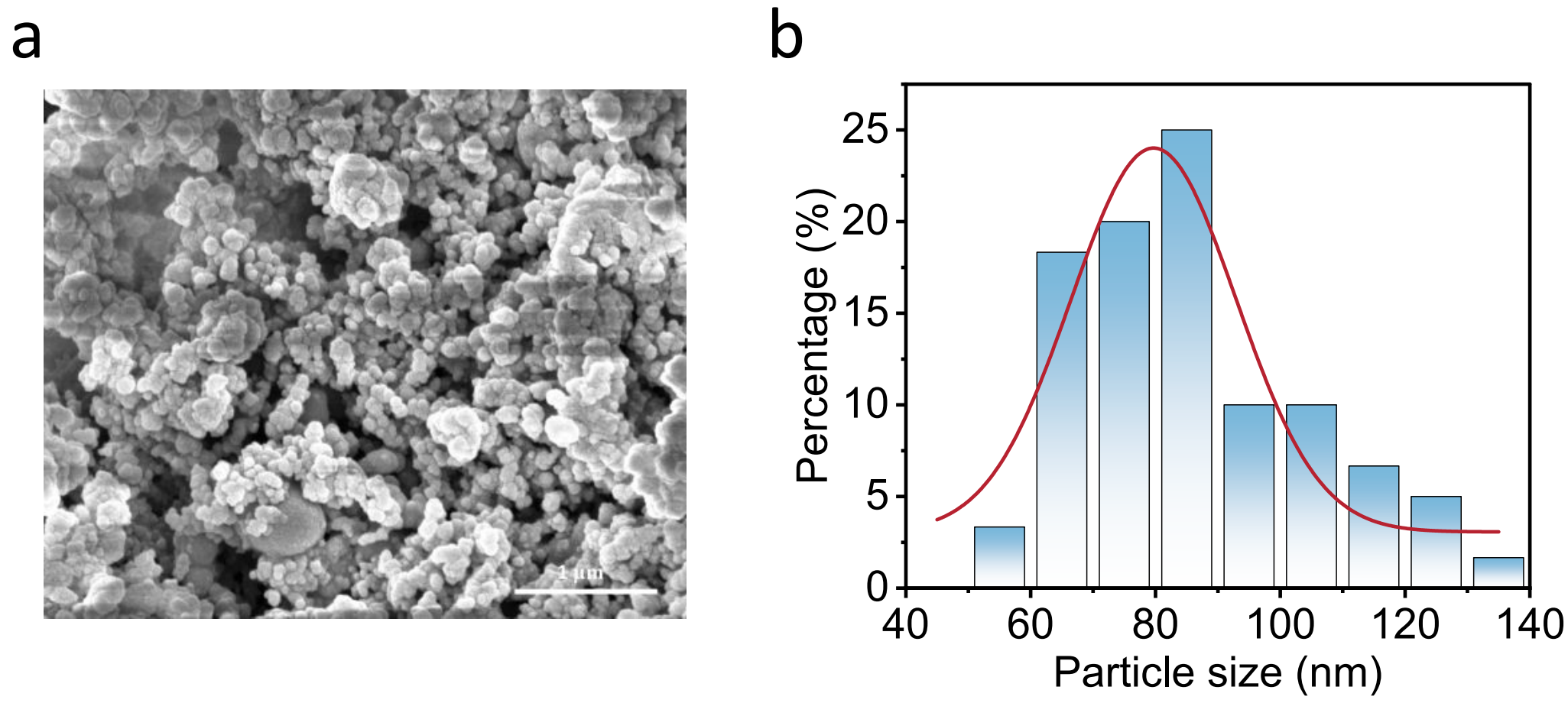


Figure S4. (a) SEM image of dried PAA-MgACCP particles. (b) Particle-size distribution map obtained by SEM.

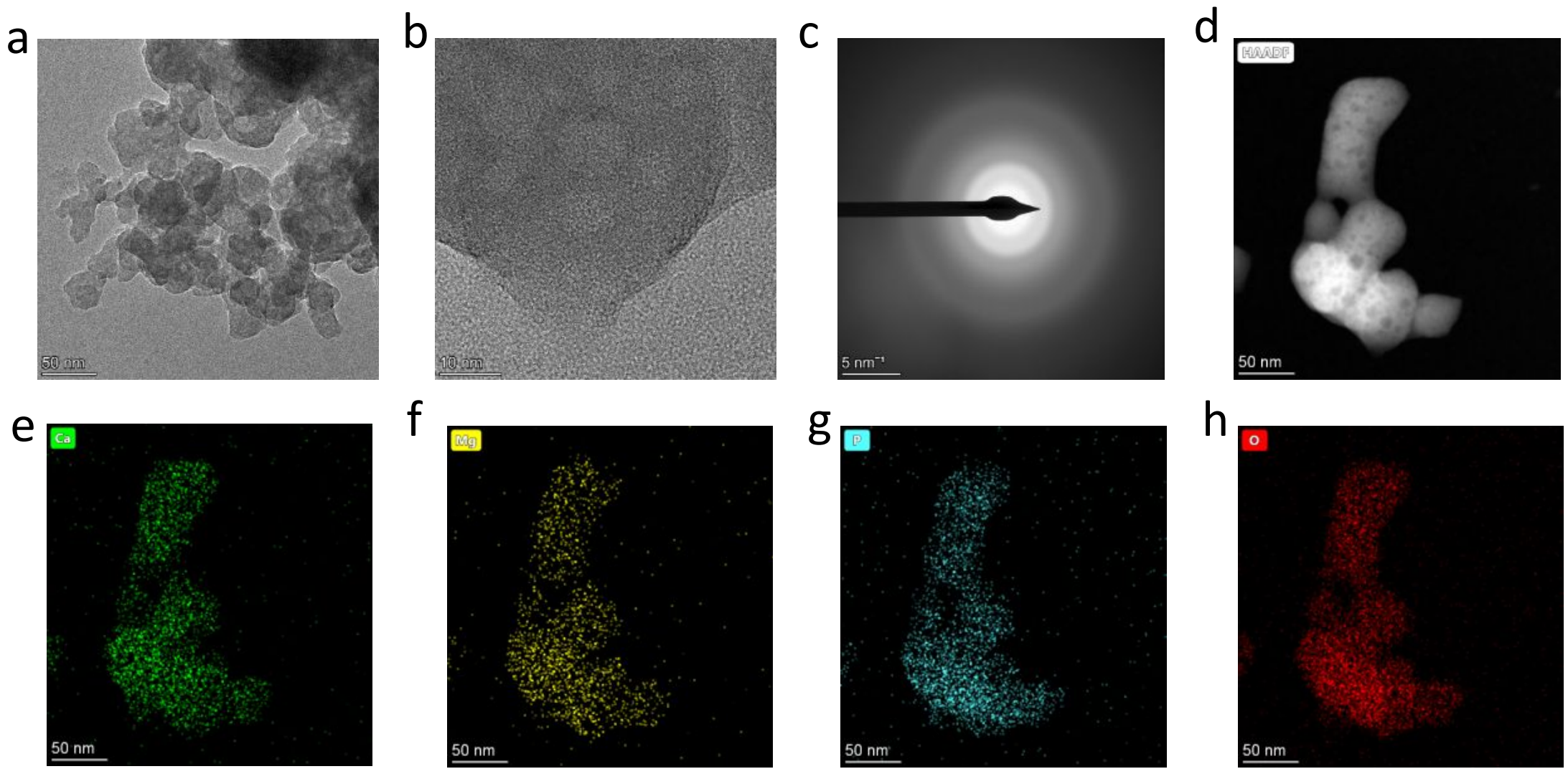


Figure S5. (a) TEM and (b) HRTEM images showing the morphology of MgACCP gel. (c) SAED pattern indicating the amorphous nature of the sample. (d) HAADF-STEM image and (e-h) corresponding EDS elemental mapping of Ca, Mg, P, and O, demonstrating the uniform distribution of elements.

Comparatively, the mineral gels maintain dispersity both before and after the incorporation of PAA. The introduction of PAA led to less distinct particle boundaries, resulting in the formation of a continuous and dense network. Notably, the SAED patterns consistently exhibited diffuse halo rings, confirming that the addition of PAA does not induce the crystallization of amorphous calcium carbonate, thereby preserving its stable amorphous state.

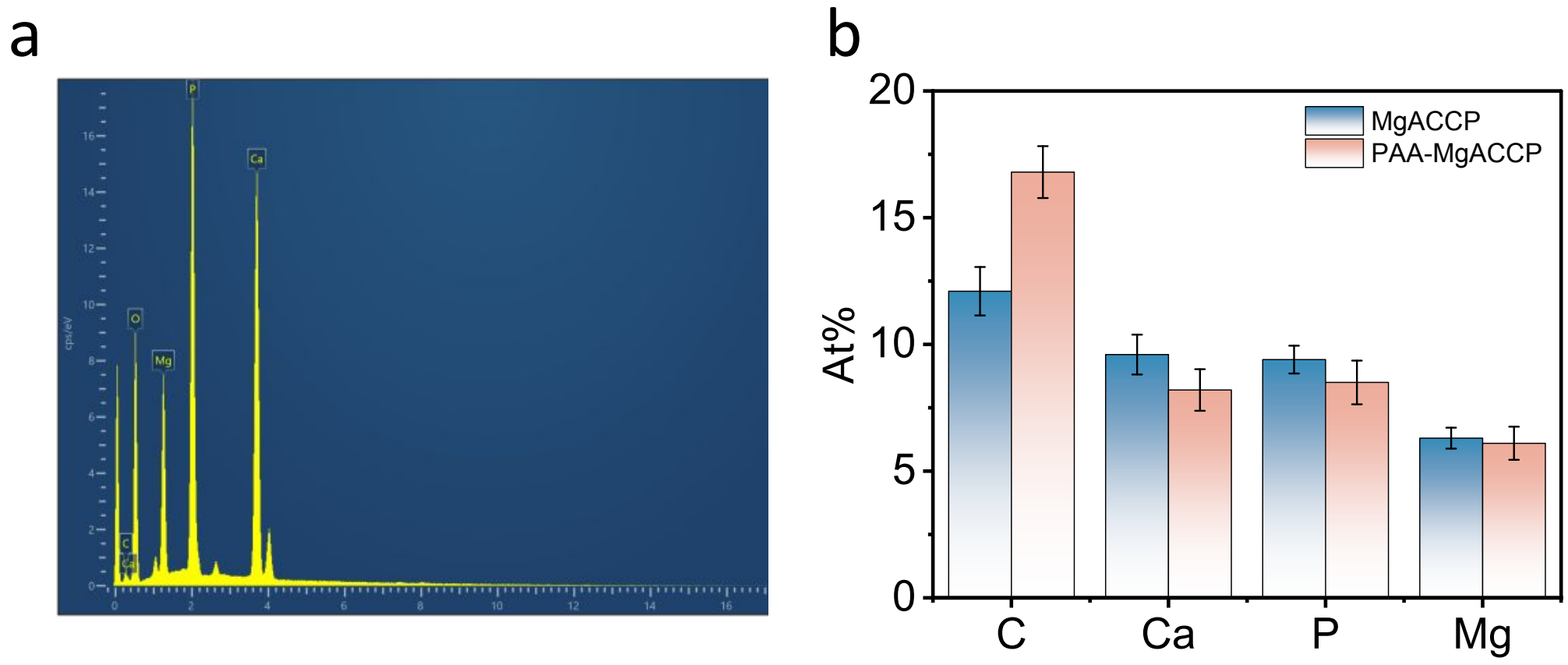


Figure S6. (a) Representative EDX spectrum of the MgACCP-based sample. (b) Atomic percentages (At%) of C, Ca, P, and Mg for MgACCP and PAA-MgACCP. The higher C content in PAA-MgACCP is attributed to the presence of PAA.

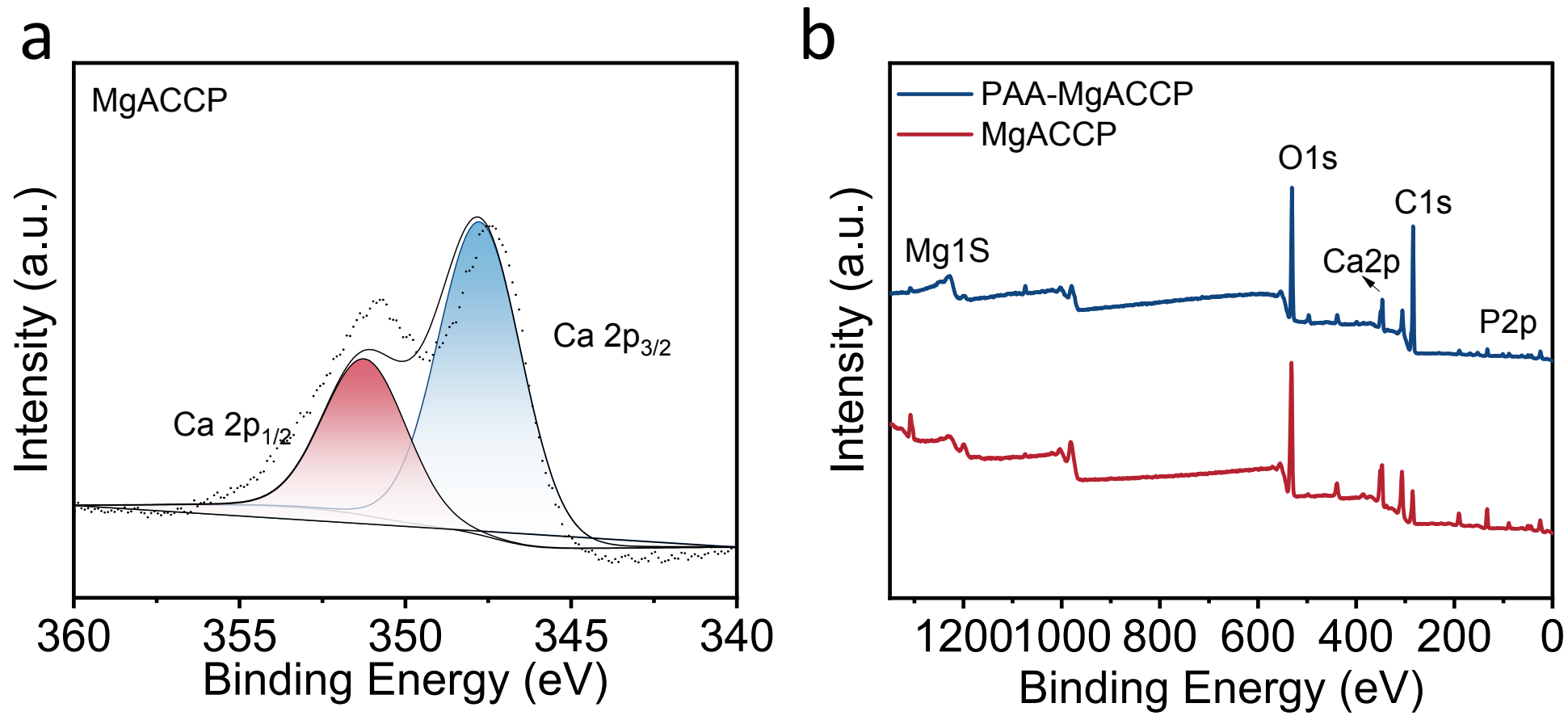


c

| Sample | Component | Ca $2p_{3/2}$ / eV | Ca $2p_{1/2}$ / eV | FWHM / eV | Area fraction |
|---|---|---|---|---|---|
| MgACCP | Ca-I | 347.1479 | 350.6179 | 1.7840 | 35.51% |
| MgACCP | Ca-II | 348.4663 | 351.9363 | 3.2000 | 64.49% |
| PAA-MgACCP | Ca-main | 347.3474 | 350.8174 | 1.7799 | 100% |
| MgACCP control | Ca-average | 347.7508 | 351.2208 | 2.9509 | 100% |

Figure S7. (a) Forced single-doublet fitting of the MgACCP Ca 2p spectrum. (b) XPS survey spectra of MgACCP and PAA-MgACCP. (c) Summary of the corrected binding energies, FWHM values, and area fractions obtained from the constrained Ca 2p fitting.

Two constrained Ca 2p doublets are required to describe the broader Ca 2p envelope of MgACCP. However, to further verify the Ca 2p fitting strategy, MgACCP was additionally fitted using a forced single constrained Ca 2p doublet. The forced single-doublet model resulted in a broadened Ca 2p envelope with a FWHM of 2.95 eV. This broad fitted feature represents an averaged description of the heterogeneous Ca coordination environments in pristine MgACCP and does not fully capture the asymmetric peak shape.

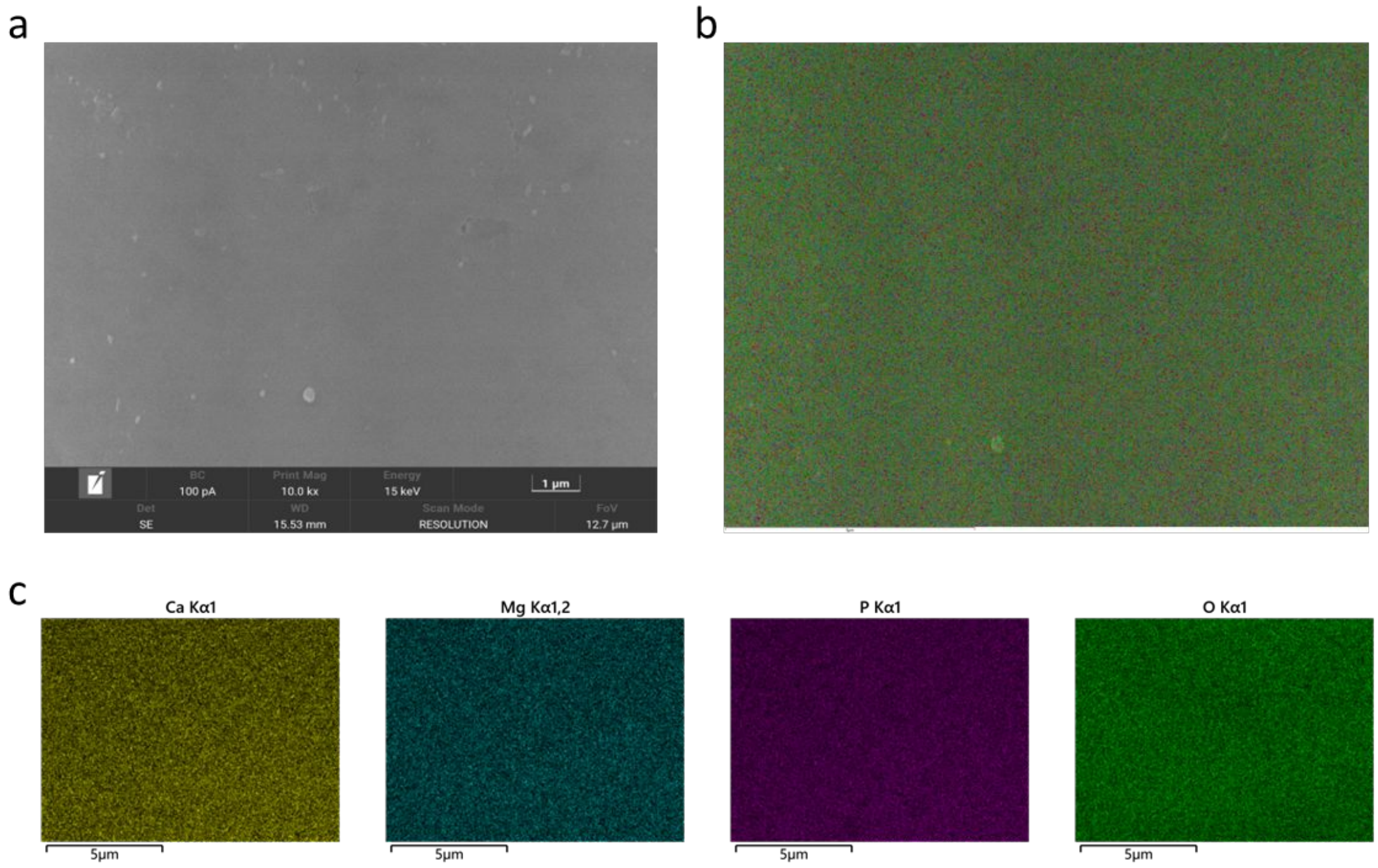


Figure S8. (a) SEM surface images of PAA-MgACCP tablets compressed at 800 MPa. Scale bar, 1 μm. (b-c) elemental mapping images of PAA-MgACCP tablets. Scale bar, 5 μm.

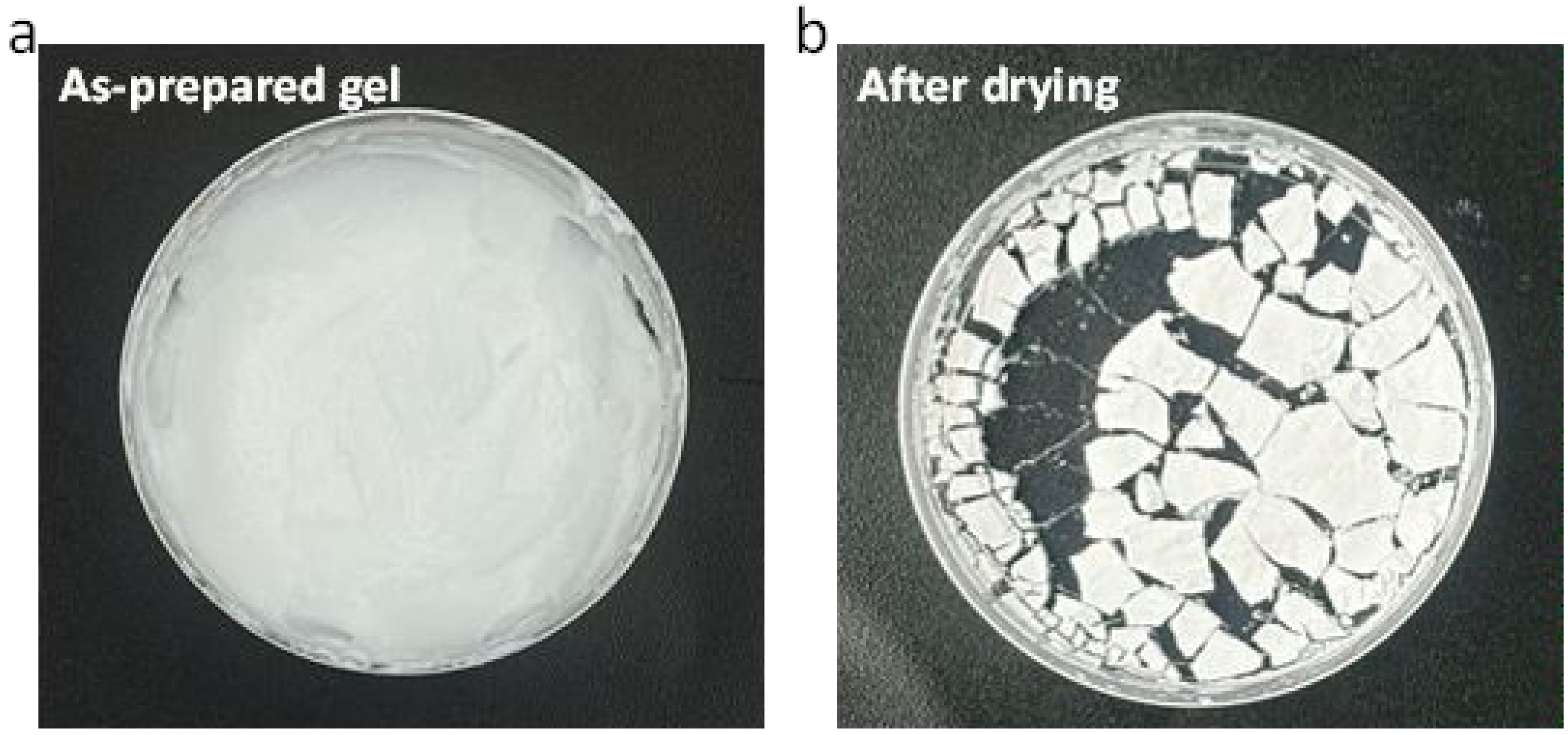


Figure S9. Photographs of (a) the as-prepared PAA-MgACCP gel in a circular mold and (b) the resulting cracked solid after direct drying at 80 °C.

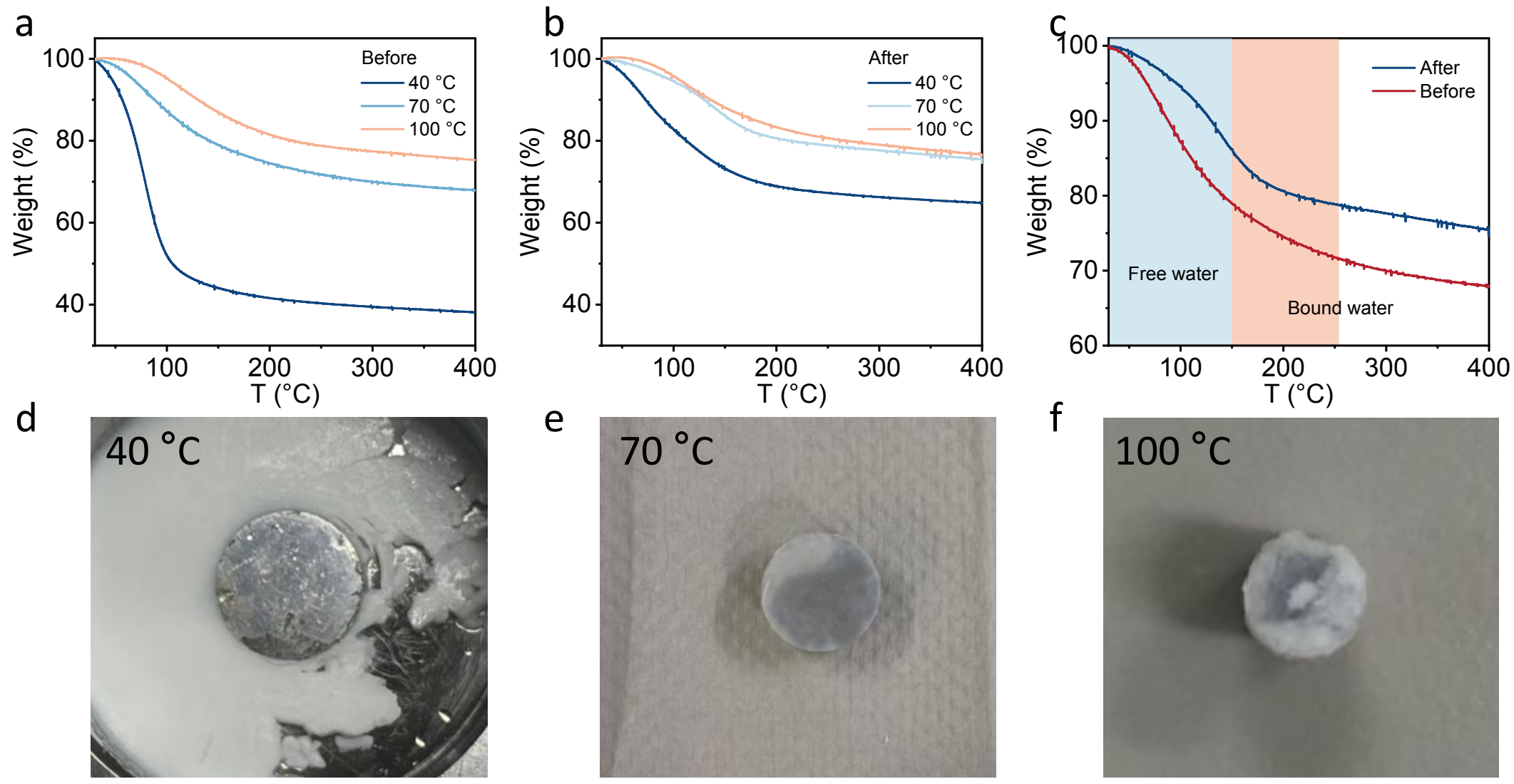


Figure S10. Effect of moisture content on the tableting process and the thermal behavior of the samples. (a) TG curves of gel powders. (b) TG curves of the corresponding tablets after pressing. (c) Dehydration-induced weight loss before and after pressing. (d–f) Representative photographs of tablets prepared from powders dried at 40 °C, 70 °C, 100 °C, respectively.

The total water content was calculated from the mass loss in the 50-250 °C range. High water content caused excessive flowability during compaction, so that the material readily migrated within the die and flowed out from the edge, making it difficult to maintain a stable geometry and to form an intact and dense tablet. In contrast, insufficient water limited the flowability and plasticity of the powder during pressing, preventing the particles from fully rearranging and establishing uniform contact. As a result, the obtained tablets showed a rough and non-uniform surface. Among the three conditions, the powder dried at 70 °C exhibited the best shaping performance. These results demonstrate that water plays a crucial role in regulating powder flowability, plasticity, and interparticle fusion during compaction, and that an optimal moisture window exists in this system.

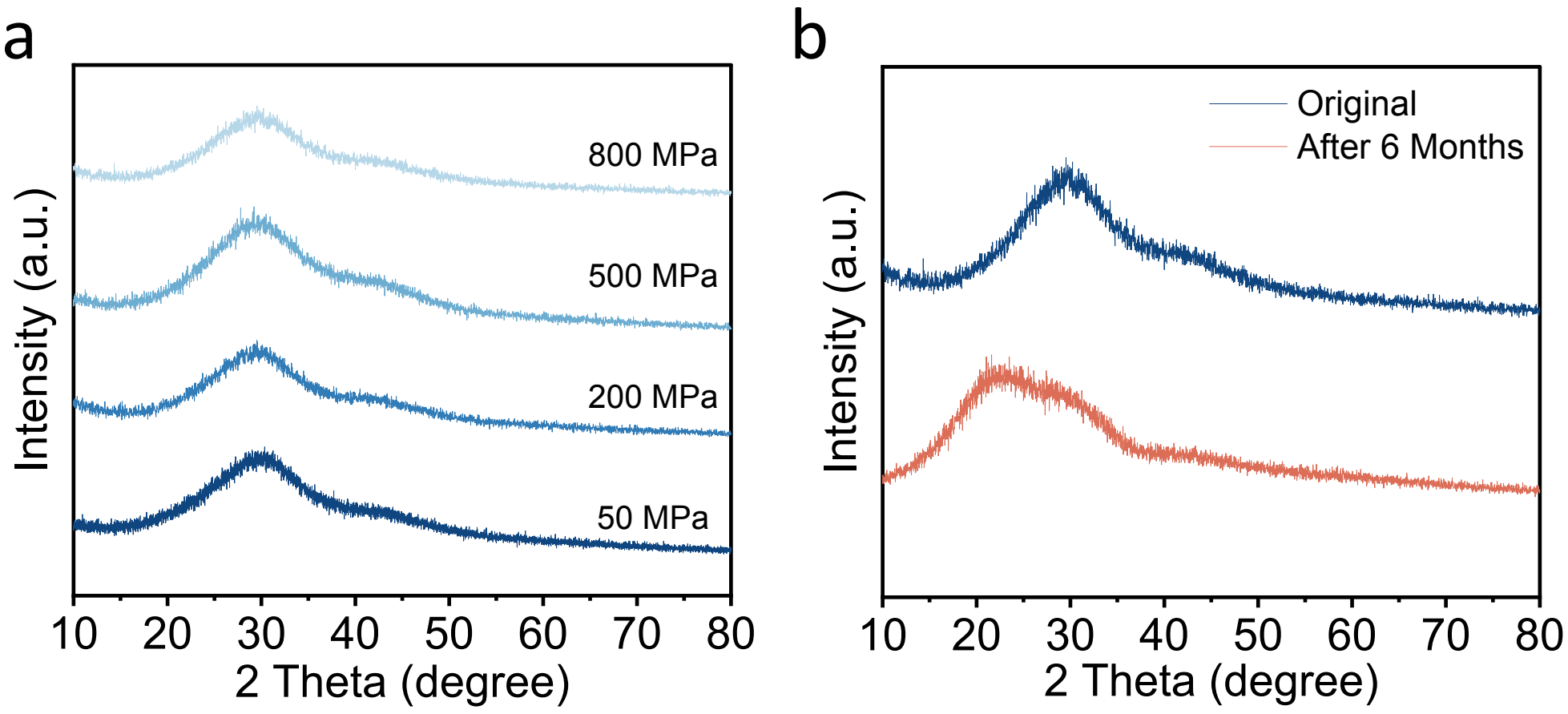


Figure S11. (a) XRD patterns of PAA-MgACCP tablets compressed at different pressures. (b) XRD patterns after 6 months of storage, with no detectable crystallization.

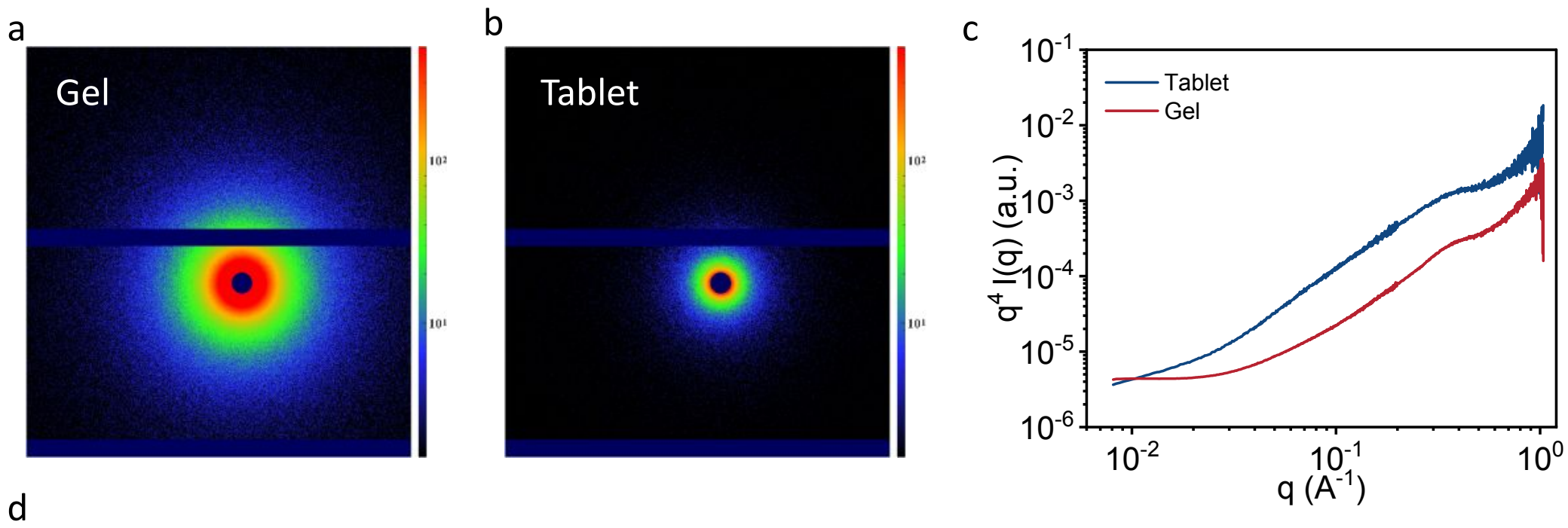


d

| Range | q/A $^{-1}$ | α |
|---|---|---|
| Low-q | 0.012-0.030 | Tablet: 2.96 |
| | | Gel: 3.79 |
| Mid-q | 0.05-0.2 | Tablet: 2.09 |
| | | Gel: 2.36 |
| High-q | 0.3-0.8 | Tablet: 2.97 |
| | | Gel: 2.56 |

Figure S12. 2D SAXS images of (a) PAA-MgACCP gel and (b) PAA-MgACCP tablet. (c) Porod representation of the SAXS profiles. (d) Summary of the power-law fitting exponents in the low-q, mid-q, and high-q regions.

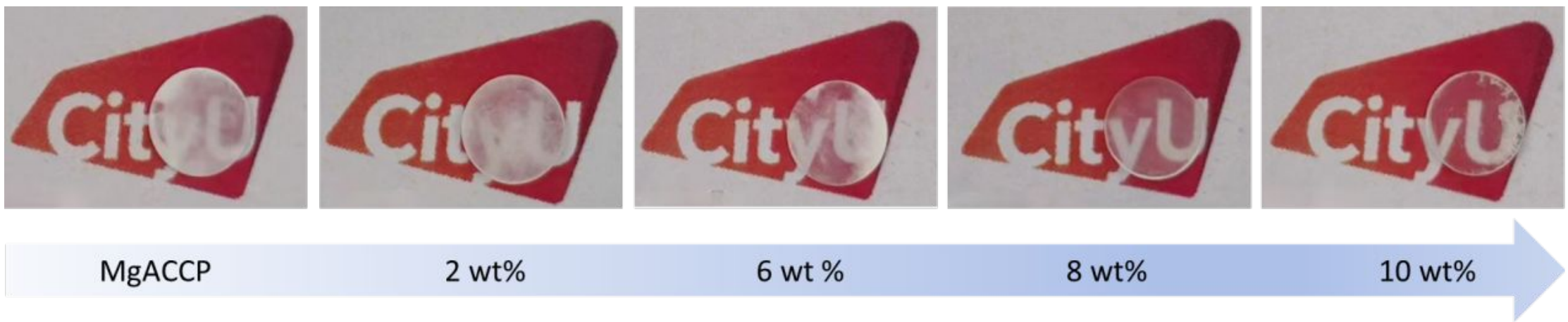


Figure S13. Photographs showing the optical transparency of tablets with PAA content of 0%, 2%, 6%, 8%, or 10 wt%.

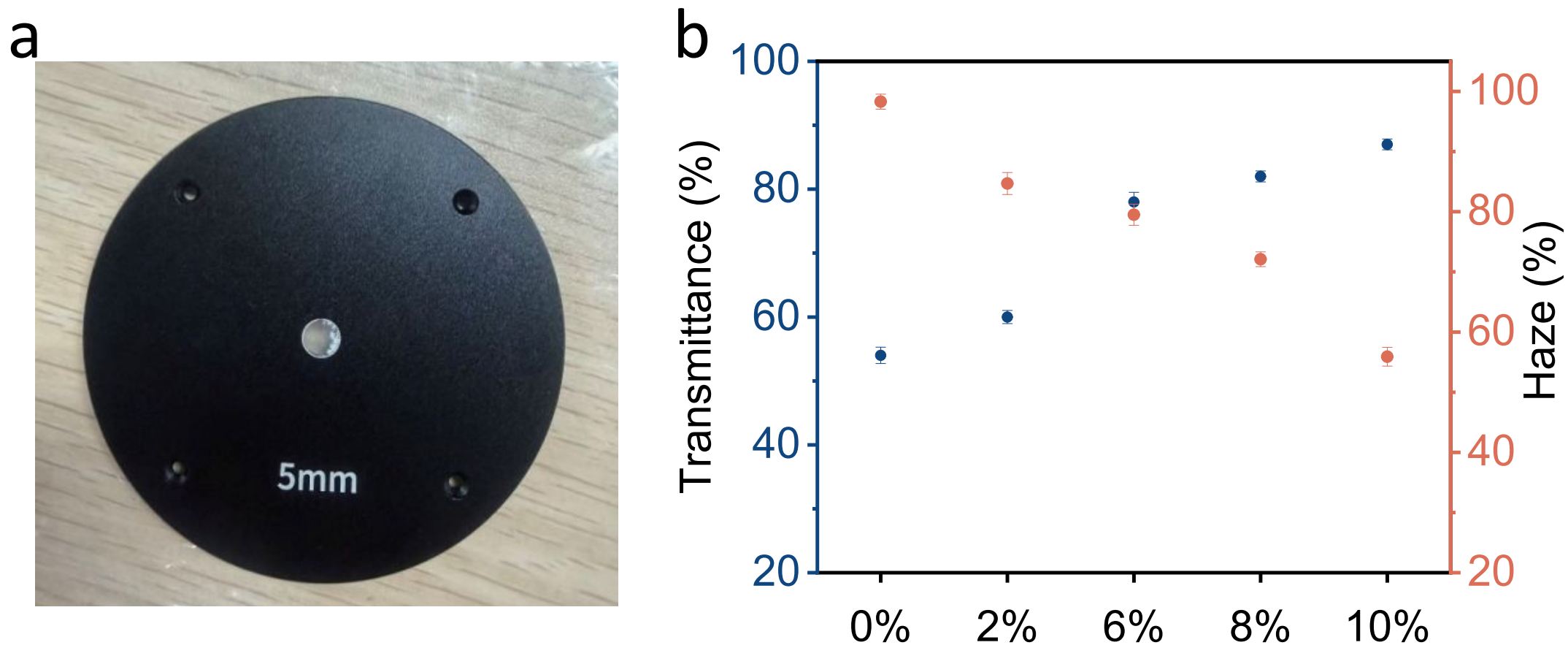


Figure S14. (a) Photograph of the selected 5 mm aperture used for optical testing. (b) Visible-light transmittance and haze of the samples as a function of PAA content.

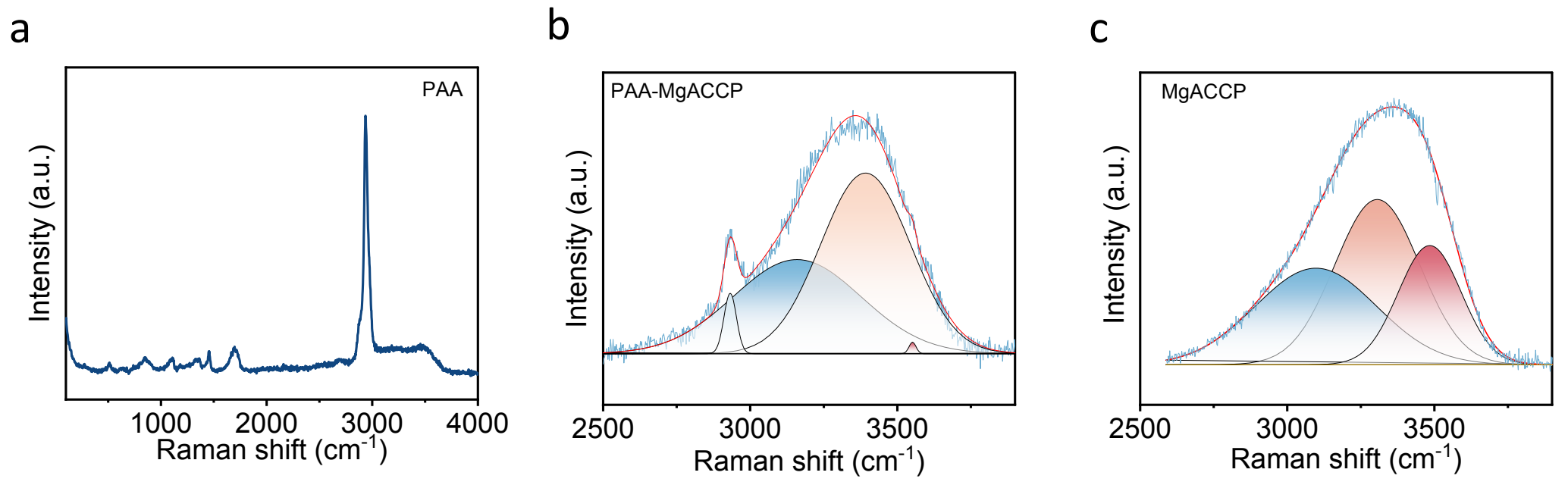


Figure S15. (a) Raman spectrum of PAA. Deconvoluted Raman spectra in the O-H stretching region (2500-3900 $cm^{-1}$) for (b) PAA-MgACCP and (c) MgACCP.

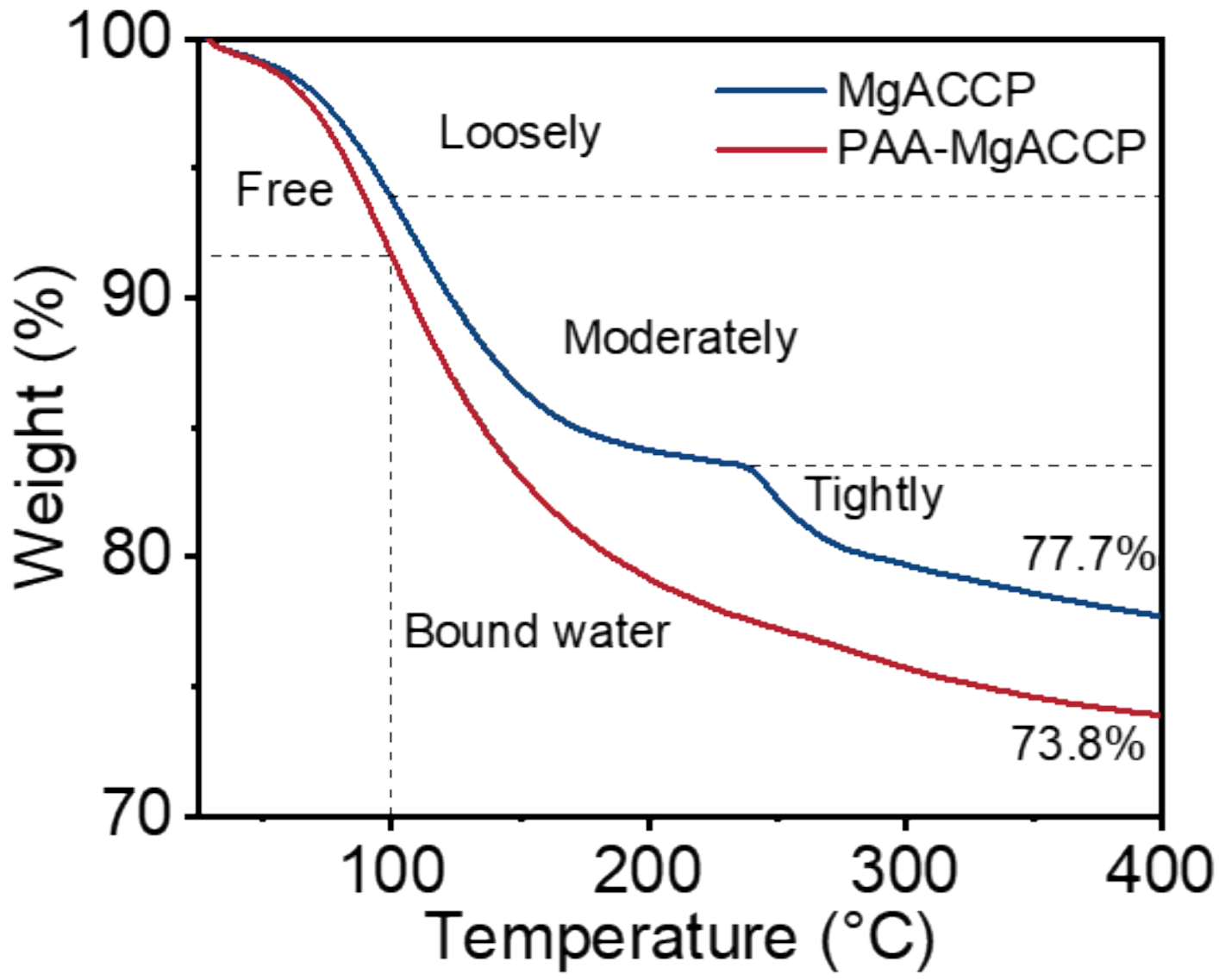


Figure S16. TG curves of MgACCP and PAA-MgACCP tablets from 25 to 400 °C.

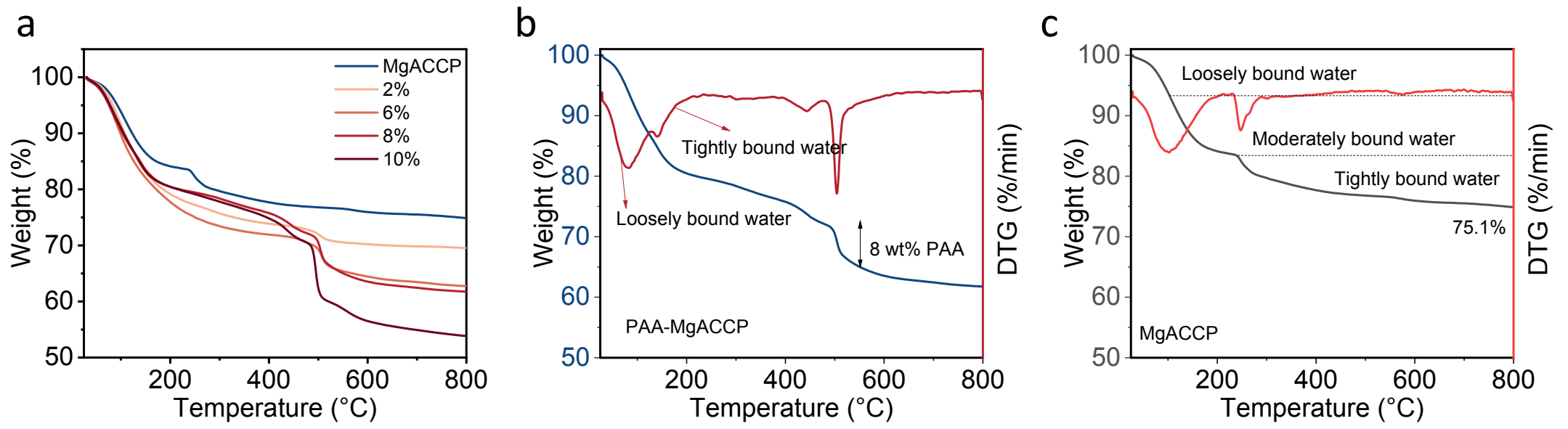


Figure S17. (a) Thermogravimetric (TG) curves of tablets from 25 °C to 800 °C. TG curves and differential thermogravimetric (DTG) curves of (b) PAA-MgACCP tablets and (c) MgACCP tablets in the temperature range of 25 °C to 800 °C.

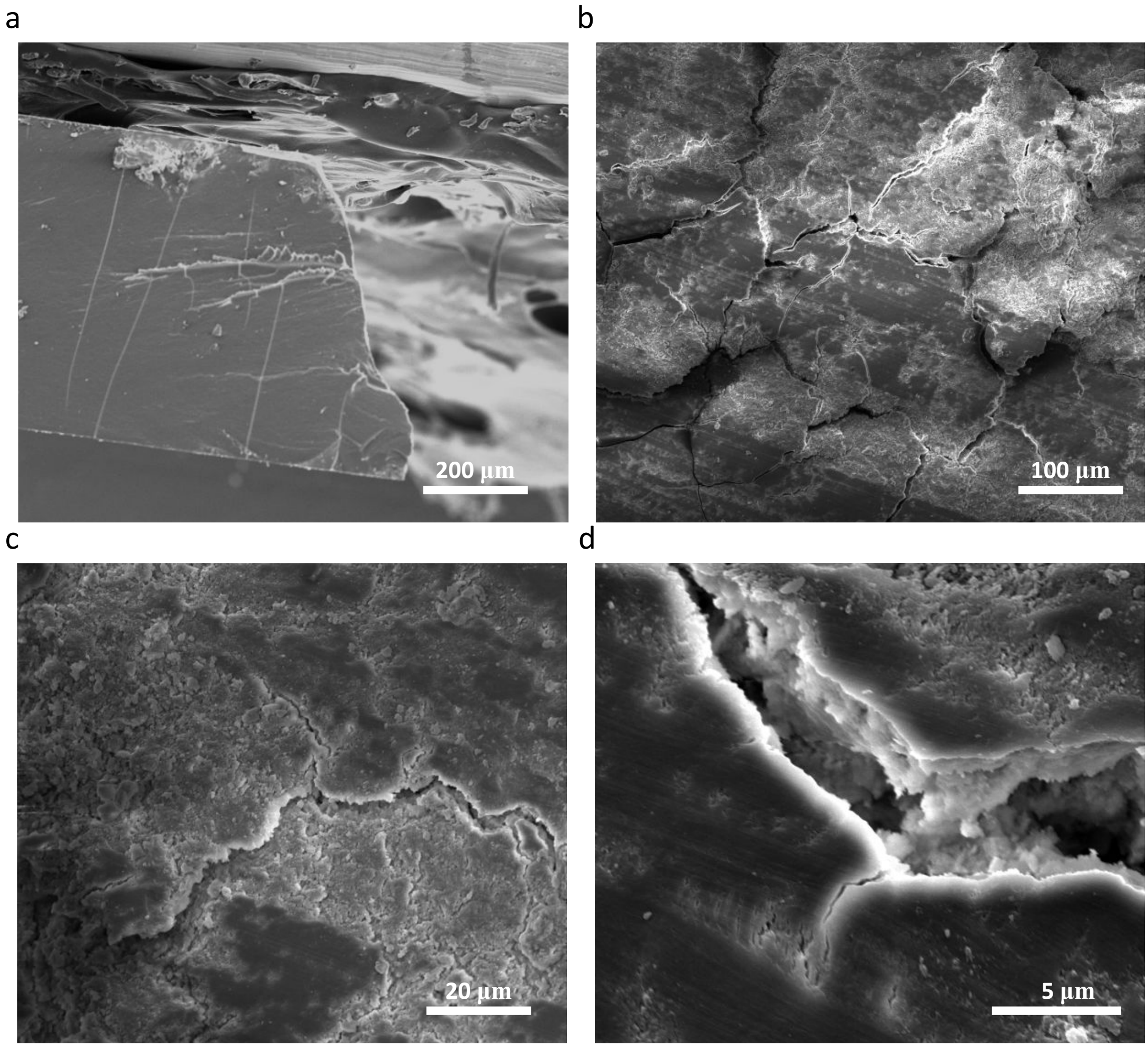


Figure S18. SEM image of the tensile fracture morphology of a PAA-MgACCP strip.

The rough and tortuous fracture morphology suggests that tensile failure does not occur through simple brittle cleavage, but rather through a progressive damage process involving crack deflection, interfacial separation, and local structural rearrangement. Such a failure mode is consistent with the incorporation of PAA, which provides deformability and stress-buffering capability to the otherwise rigid inorganic framework.

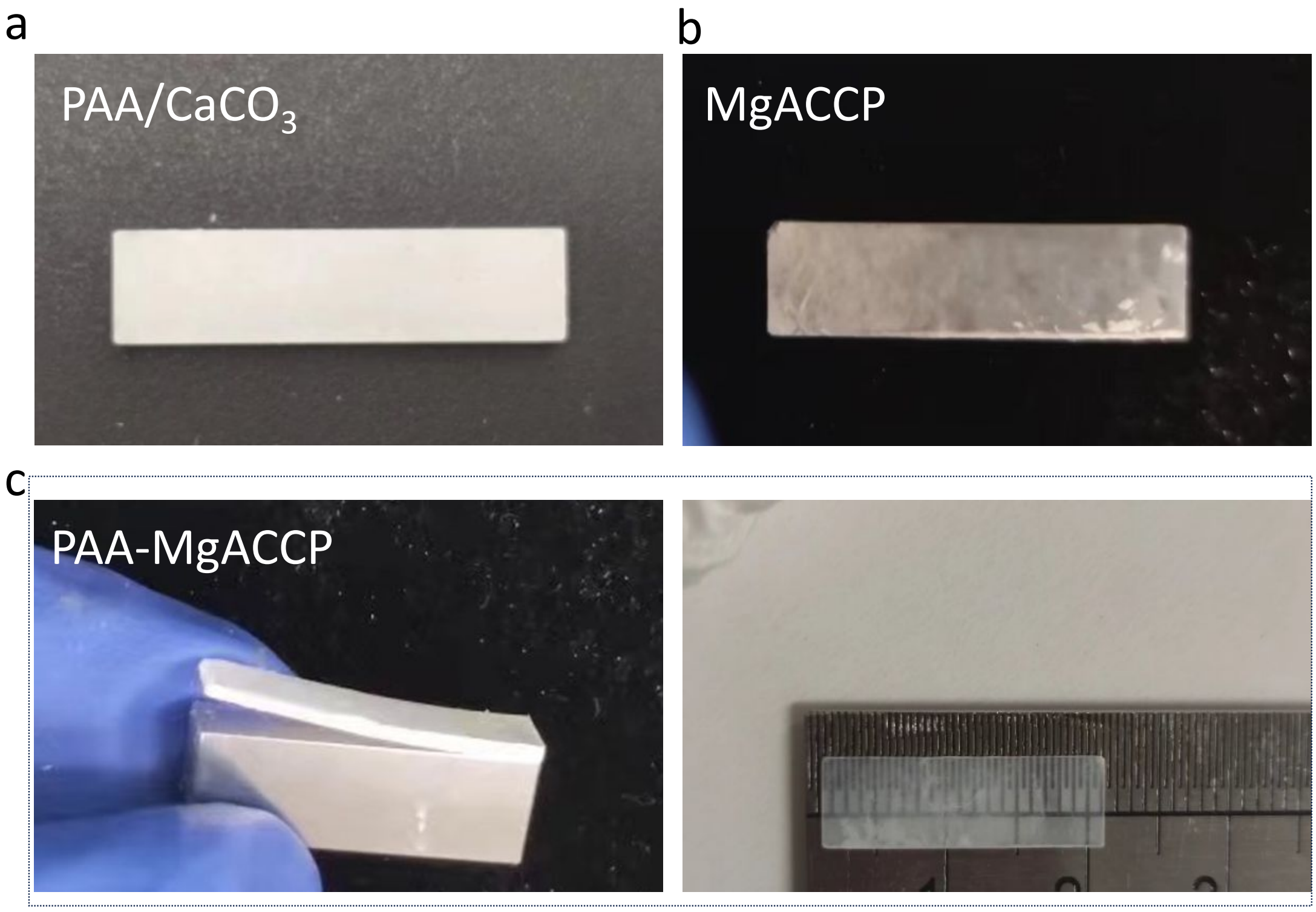


Figure S19. (a) Photographs of PAA/$CaCO_3$, (b) MgACCP, and (c) flexible PAA-MgACCP tablets.

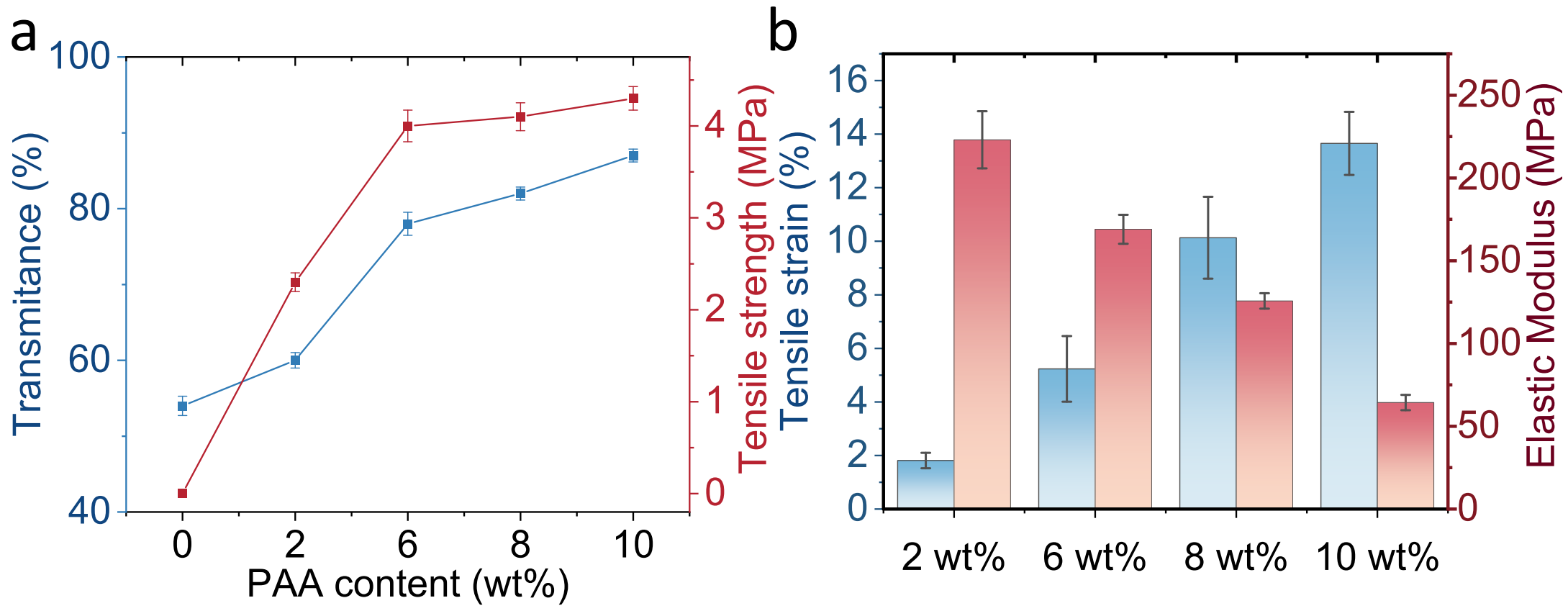


Figure S20. Trade-off between optical transmittance and tensile strength as a function of PAA content.

Although the transmittance gradually increased with increasing PAA content, the modulus decreased markedly. In addition, the tensile strength showed little change once the PAA content exceeded 6 wt%.

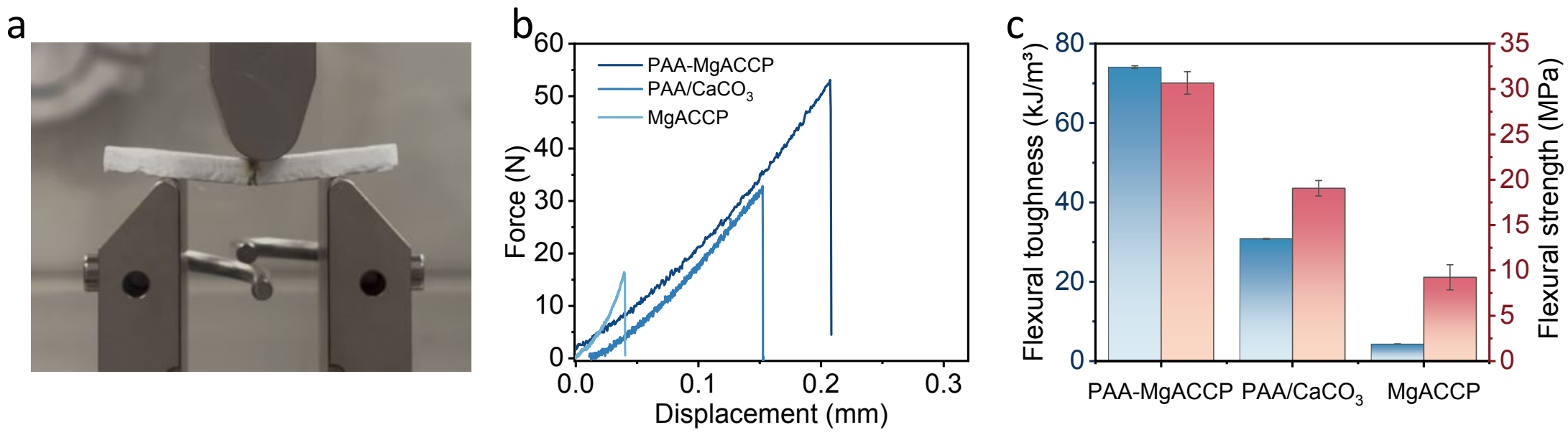


Figure S21. (a) Photograph showing the three-point bending test and deformation of the PAA-MgACCP specimen. (b) Representative force-displacement curves of PAA-MgACCP, PAA/$CaCO_3$, and MgACCP. (c) Corresponding flexural toughness and flexural strength calculated from the three-point bending tests.

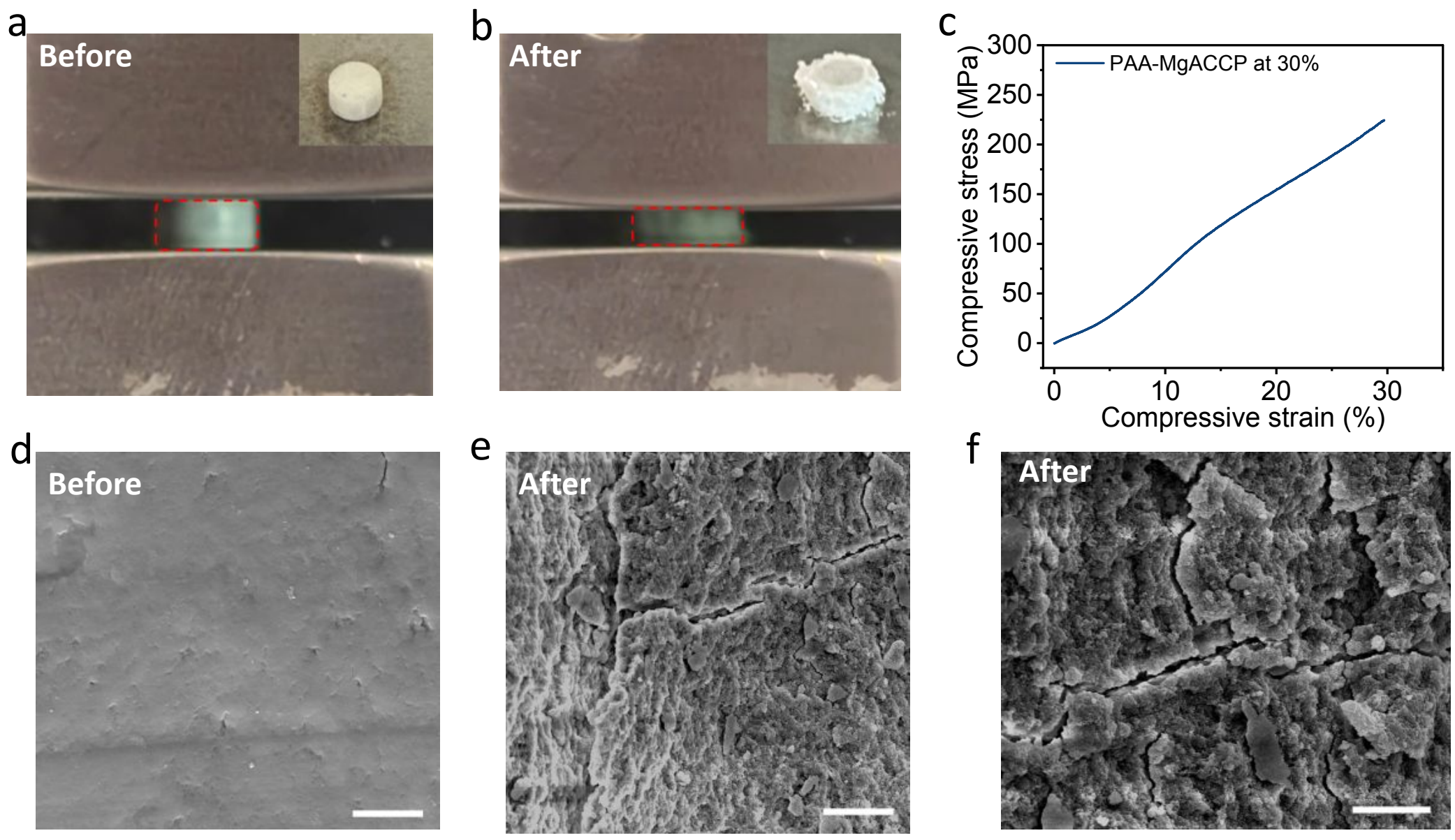


Figure S22. Mechanical response and multi-scale morphological evolution of the PAA-MgACCP tablet under uniaxial compression. (a) Digital photographs of the specimen before and (b) after compression. (c) Representative compressive stress-strain curve of the PAA-MgACCP tablet up to 30% strain. (d) SEM image of the pristine specimen. (e, f) SEM images of the surface morphology after compression at different magnifications. Scale bars: (d, e) 10 μm, (f) 5 μm.

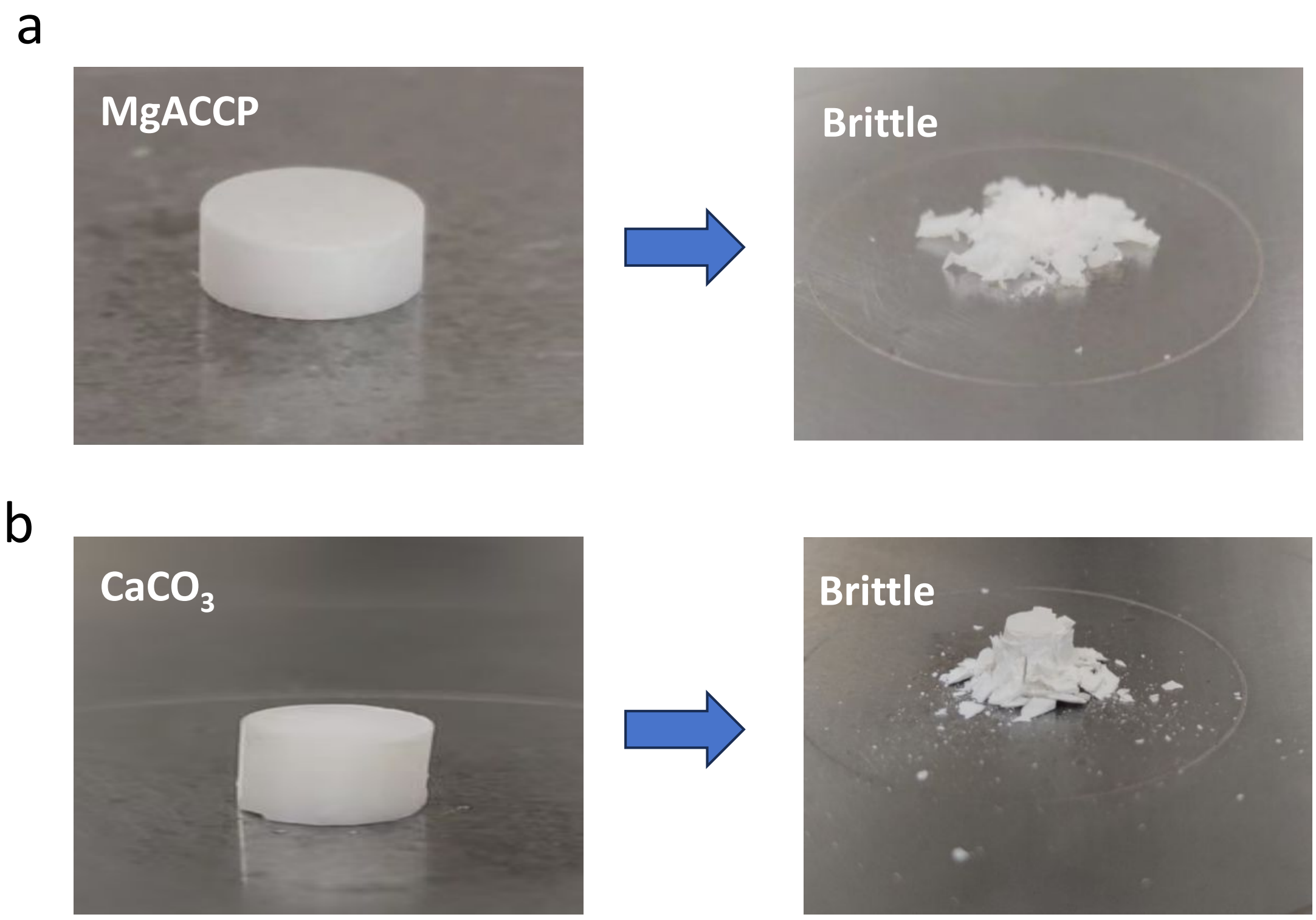


Figure S23. Photographs of (a) MgACCP and (b) PAA/$CaCO_3$ tablets before and after compression.

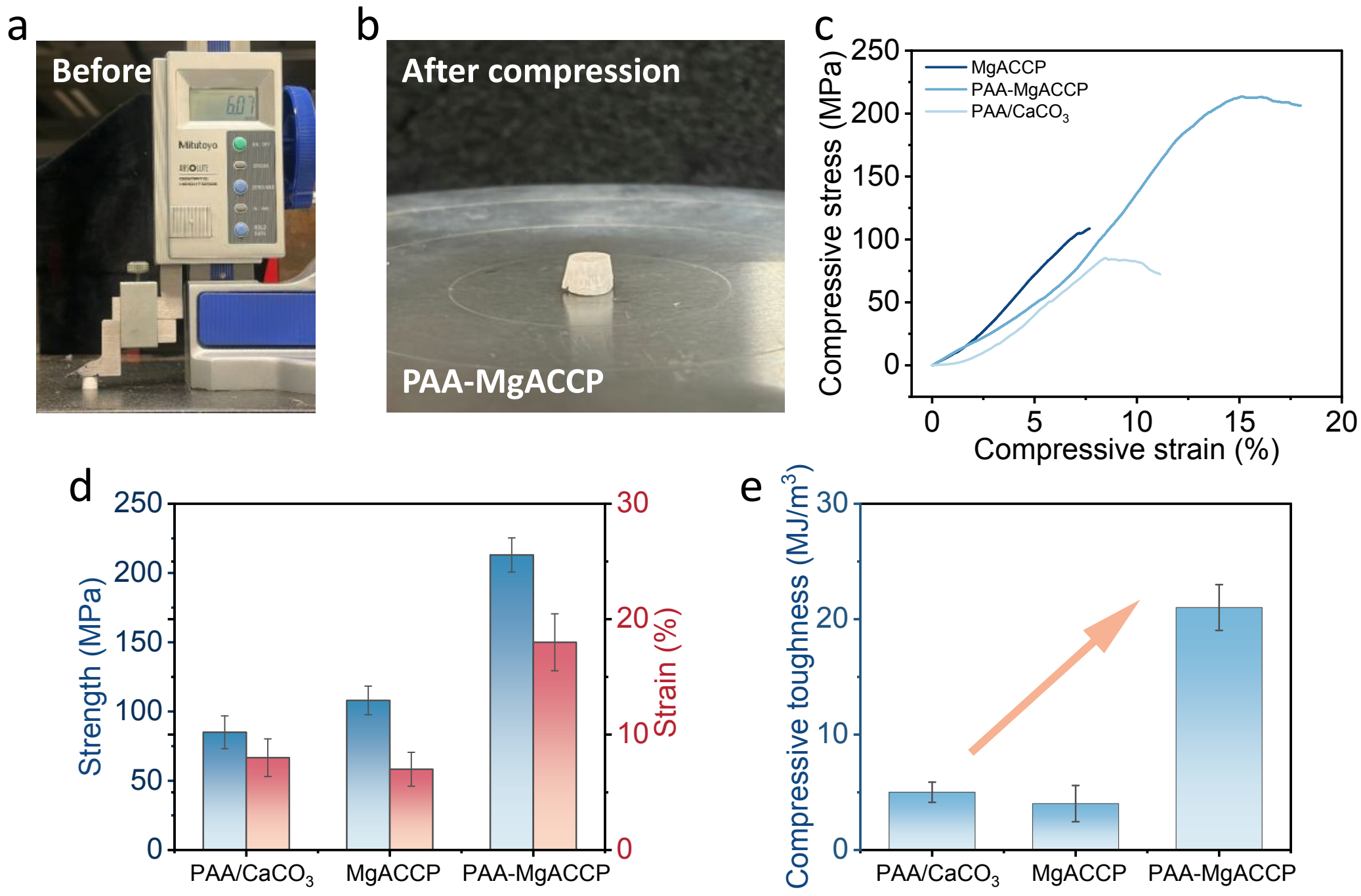


Figure S24. (a) Digital images of the PAA-MgACCP sample before and (b) after the compression test. (c) Compressive stress-strain curves of MgACCP, PAA-MgACCP, and PAA/$CaCO_3$. (d) Compressive strength and failure strain of PAA/$CaCO_3$, MgACCP, and PAA-MgACCP specimens. (e) Comparison of compressive toughness (energy density) calculated from the area under the stress-strain curves.

To further evaluate the influence of sample dimensions on the mechanical properties and address potential testing artifacts, we prepared and tested cylindrical mineral tablets with a strictly 1:1 height-to-diameter ratio. Notably, the PAA-MgACCP composite still exhibits the most outstanding mechanical performance, achieving the highest compressive strength (~213 MPa) and compressive toughness (~21 MJ/$m^3$), which are significantly superior to both PAA/$CaCO_3$ and pure MgACCP. The overall trend among the different groups remains highly consistent.

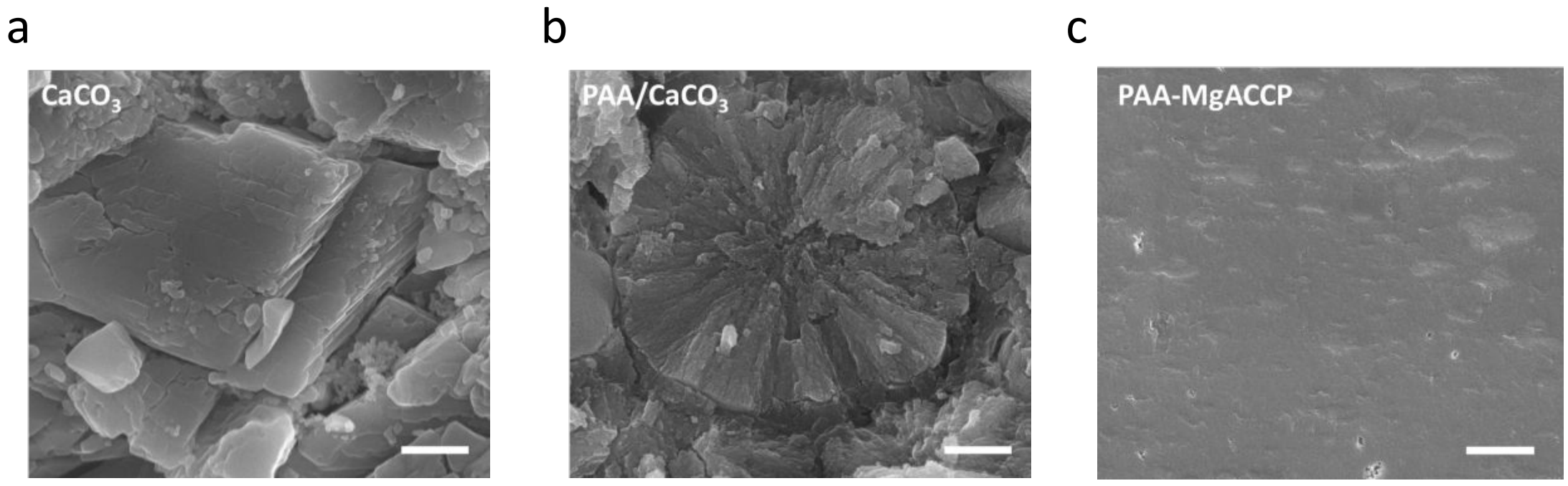


Figure S25. Cross-section SEM images of (a) $CaCO_3$, (b) PAA/$CaCO_3$, (c) PAA-MgACCP tablets. Scale bar, 1 μm.

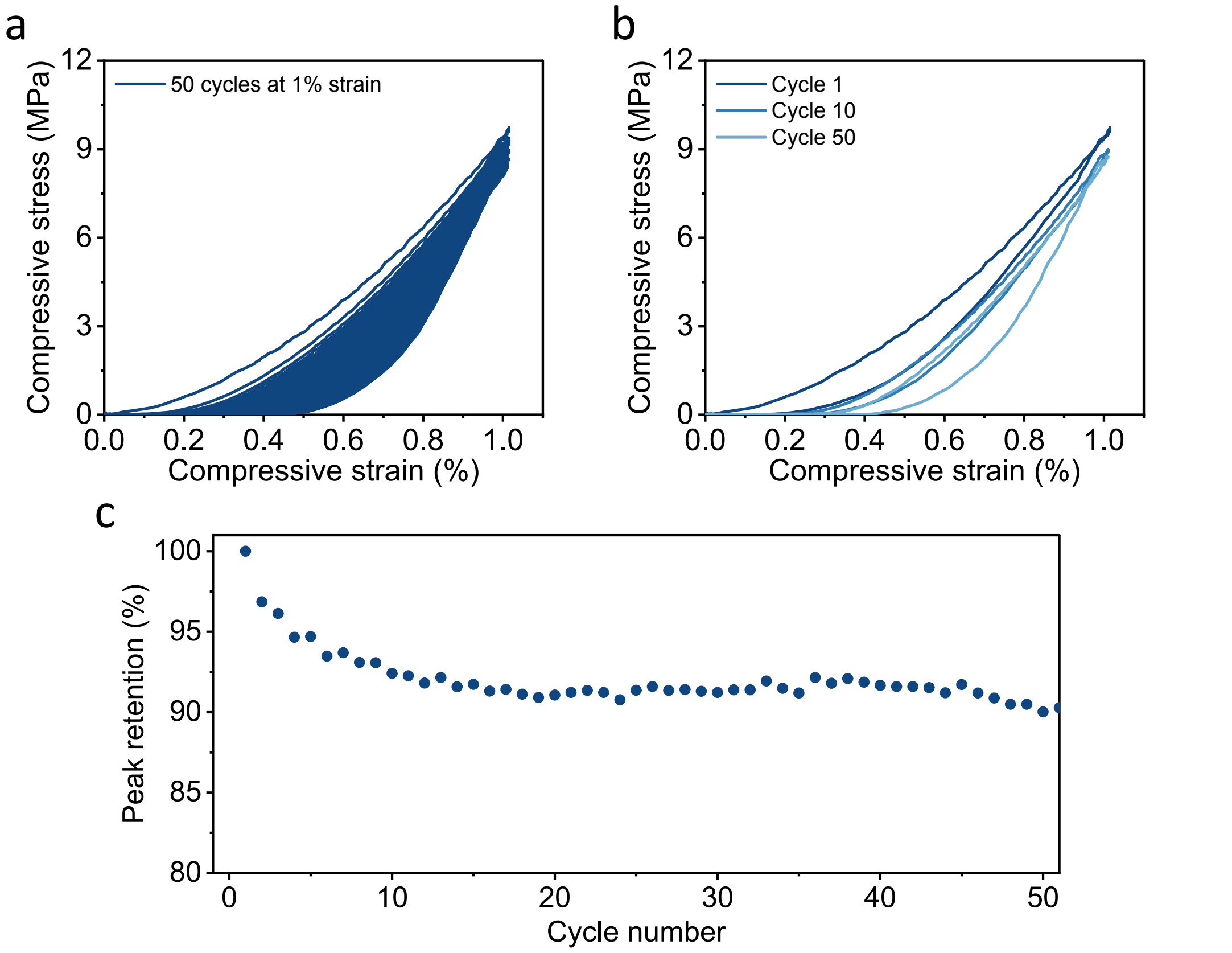


Figure S26. Low-strain cyclic compressive stability of PAA-MgACCP under 1% strain amplitude.

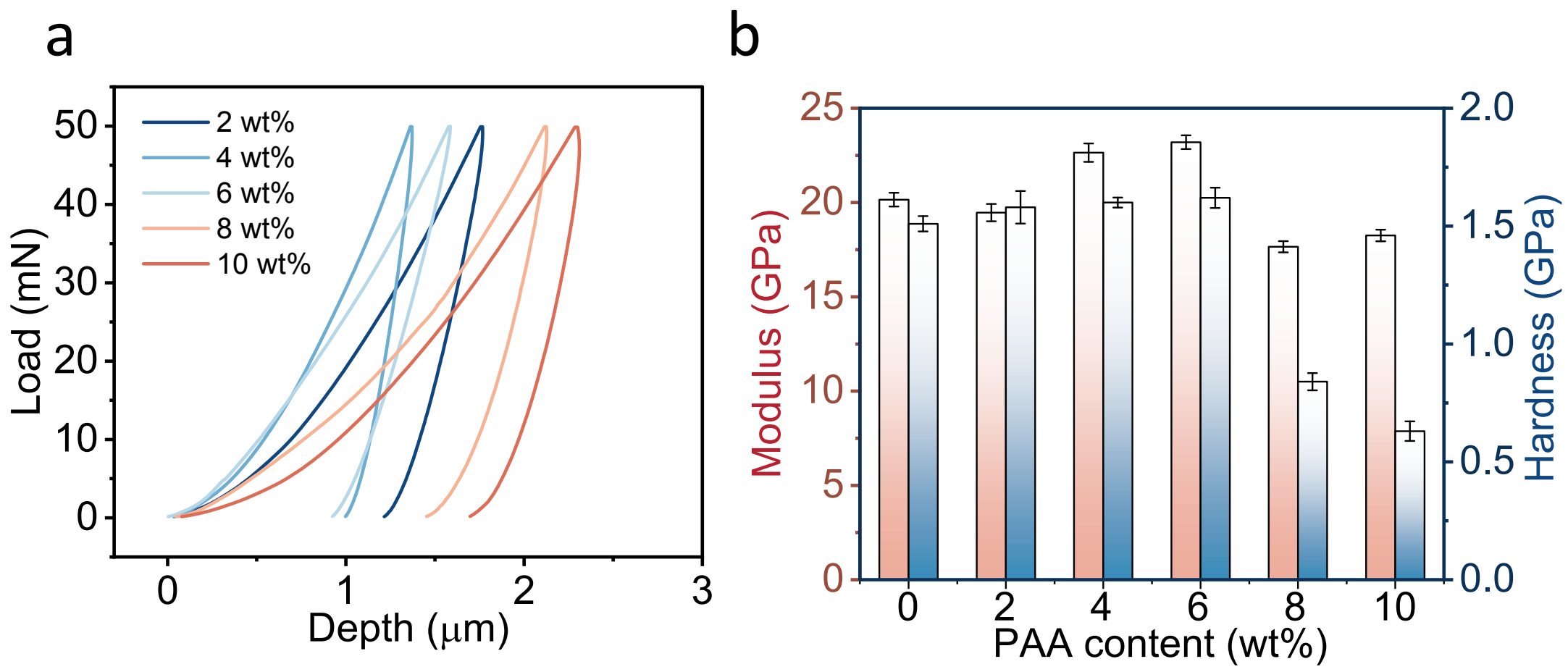


Figure S27. (a) Representative microindentation load-depth curves of PAA-MgACCP with PAA content of 2-10 wt%. (b) Corresponding indentation modulus and hardness as a function of PAA content.

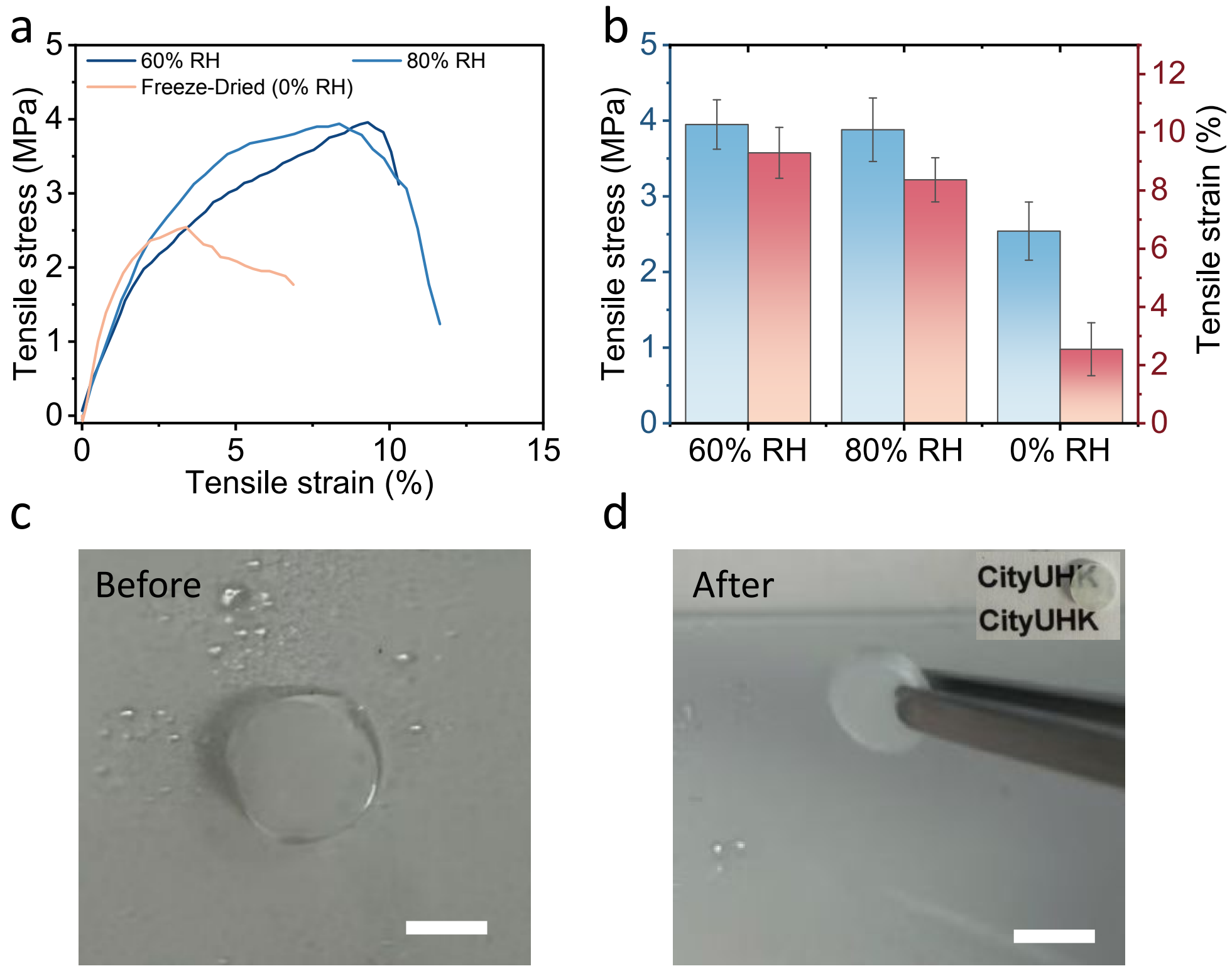


Figure S28. (a) Representative tensile stress-strain curves of PAA-MgACCP tablets under 0%, 60%, and 80% humidity conditions and (b) the corresponding tensile strength and elongation at break. (c-d) Optical images of the sample before and after water immersion, showing negligible change in appearance and morphology. Scale bar, 5 mm.

The samples at 60% RH and 80% RH exhibited similar tensile behavior, with tensile strengths of 4.0 MPa and 3.9 MPa, respectively, and tensile strains of about 9–10% and 8–9%. In contrast, the freeze-dried sample showed reduced mechanical performance, with a tensile strength of 2.5 MPa and a tensile strain of only 2.6%, indicating increased brittleness after water removal. After droplet wetting, the tablet retained its overall shape and integrity. The samples under 60% RH and 80% RH retained relatively high optical transmittance, with values of 81% and 84%, respectively. After complete dehydration at 0% RH, the transmittance dropped to approximately 66%. Overall, these results demonstrate that the material exhibits excellent tolerance to humid environments.

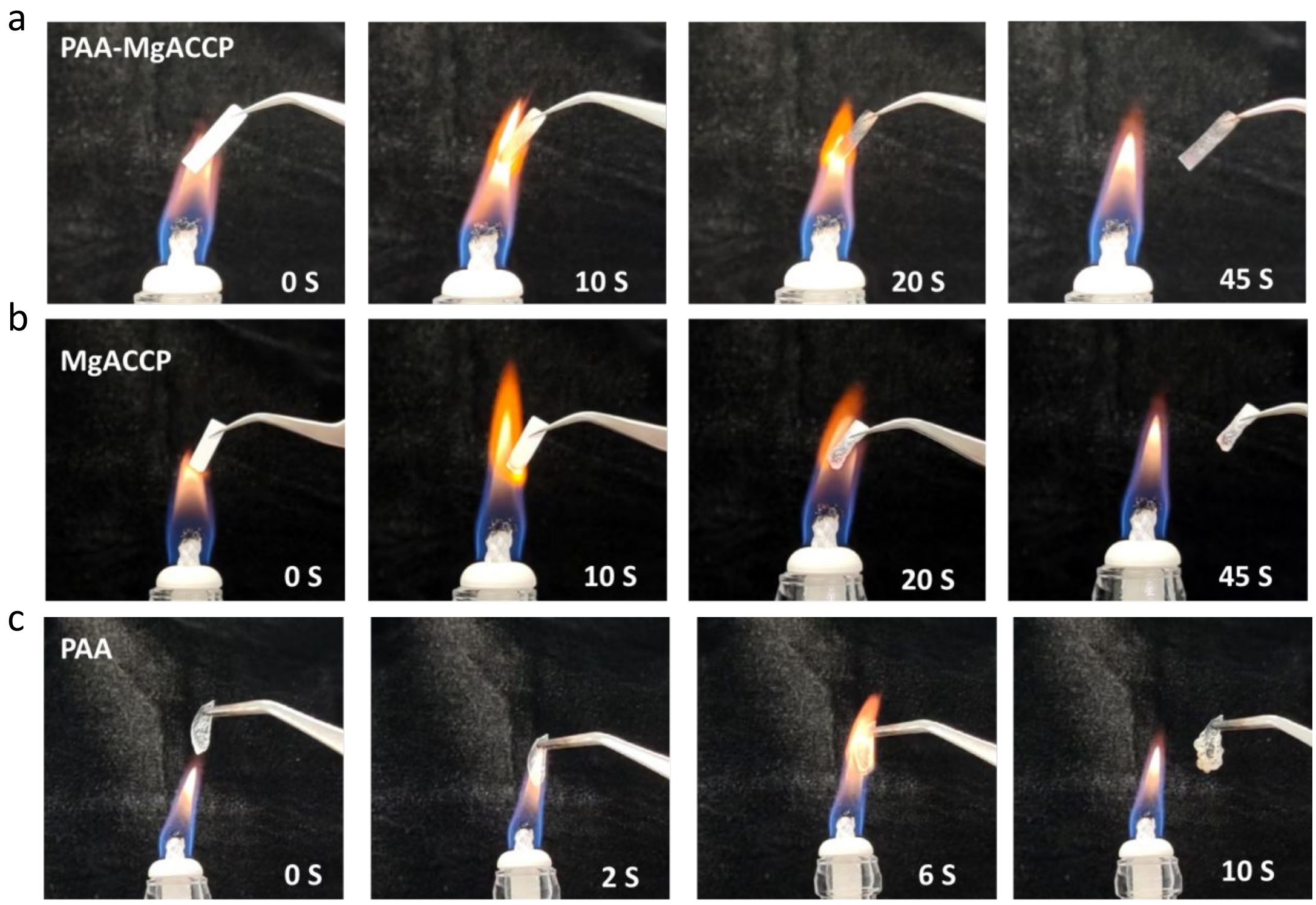


Figure S29. Qualitative flame-exposure test of (a) PAA-MgACCP, (b) MgACCP, and (c) PAA film.

The flammability of the material (20 × 5 × 1 $mm^3$) was qualitatively evaluated by direct exposure to an alcohol lamp flame. PAA-MgACCP tablet developed a typical char layer after 20 seconds of burning. It self-extinguished within 45 seconds after removal from the flame and maintained structural integrity. The pure mineral MgACCP tablet similarly displayed self-extinguishing behavior. After flame exposure, a reduction in tablet volume was observed, likely due to water evaporation and the consequent densification and shrinkage of the inorganic particles. In contrast, a pure PAA polymer film subjected to the same test showed significant combustion support within 6 seconds and expanded deformation after 10 seconds.

Supplementary Table 1: Summary of optical and mechanical properties of samples with different PAA contents.

| PAA content (wt%) | Transmittance (%) | Fracture strain (%) | Tensile strength (MPa) | Modulus (GPa) | Hardness (GPa) |
|---|---|---|---|---|---|
| 0 | 54 | N/A | N/A | 20.1 | 1.51 |
| 2 | 60 | 2 | 2.3 | 19.4 | 1.58 |
| 6 | 78 | 6 | 4.0 | 23.2 | 1.62 |
| 8 | 82 | 10 | 4.1 | 17.6 | 0.84 |
| 10 | 87 | 13 | 4.3 | 18.2 | 0.63 |

Supplementary Table 2: Summary of the relationship between indentation modulus and hardness for PAA-MgACCP and representative hybrid polymers, hybrid ceramics, biominerals, and engineered materials.

| Samples | Hardness (GPa) | E (Modulus GPa) | References |
|---|---|---|---|
| PAA-MgACCP | 1.63 | 23.20 | This work |
| PAA/$CaCO_3$ | 0.40 | 11.35 | |
| MgACCP | 1.51 | 22.15 | |
| PP | 0.08 | 1.47 | [1] |
| PVA/SWNT | 0.28 | 6.93 | [2] |
| PC/MWCNT | 0.25 | 4 | [3] |
| PMMA/CNTs | 0.19 | 5 | [4] |
| POM | 0.22 | 2.8 | [5] |
| PDMS/CNTs | 0.002 | 0.01 | [6] |
| PEEK/SCF | 0.6 | 10.73 | [7] |
| Al | 0.25 | 63.7 | [8] |
| Cu | 0.44 | 98.7 | [8] |
| Ni-Co | 1.2 | 174 | [9] |
| Fe | 1.04 | 197 | [10] |
| Cu-Sn | 1.35 | 120 | [11] |
| Concrete | 0.45 | 10 | [12] |
| Calcite ceramics | 1.64 | 53.7 | [13] |
| Dentine | 0.92 | 19.89 | [14] |
| Cortical bone | 0.89 | 12.9 | [15] |
| Cancellous bone | 0.63 | 12.7 | [15,16] |
| ACC/PAA | 1 | 22.4 | [17] |
| Al2O3/PMMA | 0.741 | 10.28 | [18] |
| HA/PLLA | 0.4 | 4.8 | [19] |

Supplementary Table 3: Comparison of organic matter and mechanical properties in biomimetic mineralization research

| System | Organic content | Key mechanical metric | Ref. |
|---|---|---|---|
| PAA-MgACCP | 2-10% | Hardness (1.6 GPa), modulus (23.20 GPa). Tensile strain (10%) | This work |
| ACC–PAA hybrid | 53% | Hardness (1 GPa), modulus (22.4 GPa). | [17] |
| CaP/CNF | 30% | Tensile strain (3.6%) | [20] |
| PAAmMBAm/ACC | 24% | Strength (3.4 MPa) | [21] |
| MNHs | 82% | Hardness (~180MPa), modulus (~2.5 GPa) | [22] |
| CPO-PU | 85% | Strength (~63 MPa), | [23] |

Supplementary Table 4: Summary of differences between PAA-MgACCP and polypropylene (PP), aluminum, dentin, and concrete in terms of $H^3/E^2$, toughness, hardness, optical transmittance and specific strength.

| | PAA-MgACCP | Polymer (PP)[1] | Metal (Al)[8] | Dentine[14] | Concrete[12] |
|---|---|---|---|---|---|
| $H^3/E^2$ | 8.05 | 0.238 | 0.00386 | 1.968 | 0.834 |
| Toughness (MJ/m$^{-3}$) | 95.3 | 0.2 | 30 | / | / |
| Hardness (GPa) | 1.63 | 0.08 | 0.25 | 0.92 | 0.45 |
| Transmittance (%) | 78 | 90 | / | / | / |
| Specific strength MPa/(g cm$^{-3}$) | 909 | 88.89 | 92.59 | 438.09 | 160.71 |

## References


(1) Bhandari, N. L.; Lach, R.; Grellmann, W.; Adhikari, R. Depth-Dependent Indentation Microhardness Studies of Different Polymer Nanocomposites. *Macromol. Symp.* **2012**, *315* (1), 44–51.

(2) Prasad, K. E.; Das, B.; Maitra, U.; Ramamurty, U.; Rao, C. N. R. Extraordinary Synergy in the Mechanical Properties of Polymer Matrix Composites Reinforced with 2 Nanocarbons. *Proc. Natl. Acad. Sci. U.S.A.* **2009**, *106* (32), 13186–13189.

(3) Nagy, P. M.; Aranyi, D.; Horváth, P.; Pötschke, P.; Pegel, S.; Kálmán, E. Nanoindentation Investigation of Carbon Nanotube–Polymer Composites. *Internet Electron. J. Mol. Des.* **2006**, No. 5, 135–143.

(4) Mammeri, F.; Teyssandier, J.; Connan, C.; Le Bourhis, E.; Chehimi, M. M. Mechanical Properties of Carbon Nanotube–PMMA Based Hybrid Coatings: The Importance of Surface Chemistry. *RSC Adv.* **2012**, *2* (6), 2462–2468.

(5) Maries, G. R. E.; Bungau, C.; Chira, D.; Costea, T.; Mosteanu, D.-E. Study on the Influence of the Grind Percentage Over the Surface Hardness and Modulus of Elasticity of Parts Made of ABS, P6.6 and POM through Nanoindentation. *Mater. Plast.* **2019**, *56* (1), 65–70.

(6) Wu, C.-L.; Lin, H.-C.; Hsu, J.-S.; Yip, M.-C.; Fang, W. Static and Dynamic Mechanical Properties of Polydimethylsiloxane/Carbon Nanotube Nanocomposites. *Thin Solid Films* **2009**, *517* (17), 4895–4901.

(7) Molazemhosseini, A.; Tourani, H.; Naimi-Jamal, M. R.; Khavandi, A. Nanoindentation and Nanoscratching Responses of PEEK Based Hybrid Composites Reinforced with Short Carbon Fibers and Nano-Silica. *Polym. Test.* **2013**, *32* (3), 525–534.

(8) Tsybenko, H.; Farzam, F.; Dehm, G.; Brinckmann, S. Scratch Hardness at a Small Scale: Experimental Methods and Correlation to Nanoindentation Hardness. *Tribol. Int.* **2021**, *163*, 107168.

(9) Yang, L.; Chen, Y.; Miller, J.; Weber, W. J.; Bei, H.; Zhang, Y. Deformation Mechanisms in Single Crystal Ni-Based Concentrated Solid Solution Alloys by Nanoindentation. *Mater. Sci. Eng. A* **2022**, *856*, 143685.

(10) Zhu, P.; Zhao, Y.; Agarwal, S.; Henry, J.; Zinkle, S. J. Toward Accurate Evaluation of Bulk Hardness from Nanoindentation Testing at Low Indent Depths. *Mater. Des.* **2022**, *213*, 110317.

(11) Li, Y.; He, K.; Liao, C.; Pan, C. Measurements of Mechanical Properties of α-Phase in Cu–Sn Alloys by Using Instrumented Nanoindentation. *J. Mater. Res.* **2012**, *27* (1), 192–196.

(12) Picker, A.; Nicoleau, L.; Burghard, Z.; Bill, J.; Zlotnikov, I.; Labbez, C.; Nonat, A.; Cölfen, H. Mesocrystalline Calcium Silicate Hydrate: A Bioinspired Route toward Elastic Concrete Materials. *Sci. Adv.* **2017**, *3* (11), e1701216.

(13) Cao, M.; Hong, W. B.; Yang, X. D.; Jia, Y. Q.; Li, L.; Wu, S. Y.; Yang, H. S.; Chen, X. M. Dense and Strong Calcite Ceramics Prepared by Room-temperature Cold Sintering Based on High-pressure-enhanced Solubility. *J. Am. Ceram. Soc.* **2023**, *106* (3), 1668–

1680.
(14) Mahoney, E.; Holt, A.; Swain, M.; Kilpatrick, N. The Hardness and Modulus of Elasticity of Primary Molar Teeth:An Ultra-Micro-Indentation Study. *J. Dent.* **2000**, *28* (8), 589–594.
(15) Ni, G. X.; Choy, Y. S.; Lu, W. W.; Ngan, A. H. W.; Chiu, K. Y.; Li, Z. Y.; Tang, B.; Luk, K. D. K. Nano-Mechanics of Bone and Bioactive Bone Cement Interfaces in a Load-Bearing Model. *Biomaterials* **2006**, *27* (9), 1963–1970.
(16) Otani, T. Quantitative Estimation of Bone Density and Bone Quality Using Acoustic Parameters of Cancellous Bone for Fast and Slow Waves. *Jpn. J. Appl. Phys.* **2005**, *44* (6S), 4578.
(17) Sun, S.; Mao, L.; Lei, Z.; Yu, S.; Cölfen, H. Hydrogels from Amorphous Calcium Carbonate and Polyacrylic Acid: Bio-Inspired Materials for "Mineral Plastics." *Angew. Chem. Int. Ed.* **2016**, *55* (39), 11765–11769.
(18) Alzarrug, F. A.; Dimitrijević, M. M.; Jančić Heinemann, R. M.; Radojević, V.; Stojanović, D. B.; Uskoković, P. S.; Aleksić, R. The Use of Different Alumina Fillers for Improvement of the Mechanical Properties of Hybrid PMMA Composites. *Mater. Des.* **2015**, *86*, 575–581.
(19) Uskokovic, P. S.; Tang, C. Y.; Tsui, C. P.; Ignjatovic, N.; Uskokovic, D. P. Micromechanical Properties of a Hydroxyapatite/Poly-l-Lactide Biocomposite Using Nanoindentation and Modulus Mapping. *J. Eur. Ceram. Soc.* **2007**, *27* (2–3), 1559–1564.
(20) Yao, J.; Fang, W.; Guo, J.; Jiao, D.; Chen, S.; Ifuku, S.; Wang, H.; Walther, A. Highly Mineralized Biomimetic Polysaccharide Nanofiber Materials Using Enzymatic Mineralization. *Biomacromolecules* **2020**, *21* (6), 2176–2186.
(21) Milovanovic, M.; Mihailowitsch, L.; Santhirasegaran, M.; Brandt, V.; Tiller, J. C. Enzyme-Induced Mineralization of Hydrogels with Amorphous Calcium Carbonate for Fast Synthesis of Ultrastiff, Strong and Tough Organic–Inorganic Double Networks. *J. Mater. Sci.* **2021**, *56* (27), 15299–15312.
(22) Tan, Y.; Wang, Y.; Zhang, P.; Li, J.; Wang, F.; Shan, L.; Guo, J.; Wang, Z.; Liu, J. Bioinspired Hierarchical Hydrogels Engineered with Extreme Impact Resistance. *Adv. Funct. Mater.* **2025**, *35* (51), e08034.
(23) Cao, W.; Wu, L.; Zhai, Y.; Ghaffar, S.; Jiang, Y. Tough, Highly Transparent, and Wear Resistant Polyurethane–Calcium Phosphite Oligomer Organic–Inorganic Hybrid Nanocomposite. *ACS Appl. Polym. Mater.* **2024**, *6* (9), 5080–5088.
